# Lysosomal lipid peroxidation regulates tumor immunity

**DOI:** 10.1172/JCI164596

**Published:** 2023-04-17

**Authors:** Monika Bhardwaj, Jennifer J. Lee, Amanda M. Versace, Sandra L. Harper, Aaron R. Goldman, Mary Ann S. Crissey, Vaibhav Jain, Mahendra Pal Singh, Megane Vernon, Andrew E. Aplin, Seokwoo Lee, Masao Morita, Jeffrey D. Winkler, Qin Liu, David W. Speicher, Ravi K. Amaravadi

**Affiliations:** 1 Abramson Cancer Center and Department of Medicine, University of Pennsylvania, Philadelphia, Pennsylvania, USA.; 2 The Wistar Institute, Philadelphia, Pennsylvania, USA.; 3 Department of Cancer Biology, Sidney Kimmel Cancer Center, Thomas Jefferson University, Philadelphia, Pennsylvania, USA.; 4 Department of Chemistry, University of Pennsylvania, Philadelphia, Pennsylvania, USA.

**Keywords:** Oncology, Cancer, Cellular immune response, Lysosomes

## Abstract

Lysosomal inhibition elicited by palmitoyl-protein thioesterase 1 (PPT1) inhibitors such as DC661 can produce cell death, but the mechanism for this is not completely understood. Programmed cell death pathways (autophagy, apoptosis, necroptosis, ferroptosis, and pyroptosis) were not required to achieve the cytotoxic effect of DC661. Inhibition of cathepsins, or iron or calcium chelation, did not rescue DC661-induced cytotoxicity. PPT1 inhibition induced lysosomal lipid peroxidation (LLP), which led to lysosomal membrane permeabilization and cell death that could be reversed by the antioxidant N-acetylcysteine (NAC) but not by other lipid peroxidation antioxidants. The lysosomal cysteine transporter MFSD12 was required for intralysosomal transport of NAC and rescue of LLP. PPT1 inhibition produced cell-intrinsic immunogenicity with surface expression of calreticulin that could only be reversed with NAC. DC661-treated cells primed naive T cells and enhanced T cell–mediated toxicity. Mice vaccinated with DC661-treated cells engendered adaptive immunity and tumor rejection in “immune hot” tumors but not in “immune cold” tumors. These findings demonstrate that LLP drives lysosomal cell death, a unique immunogenic form of cell death, pointing the way to rational combinations of immunotherapy and lysosomal inhibition that can be tested in clinical trials.

## Graphical abstract

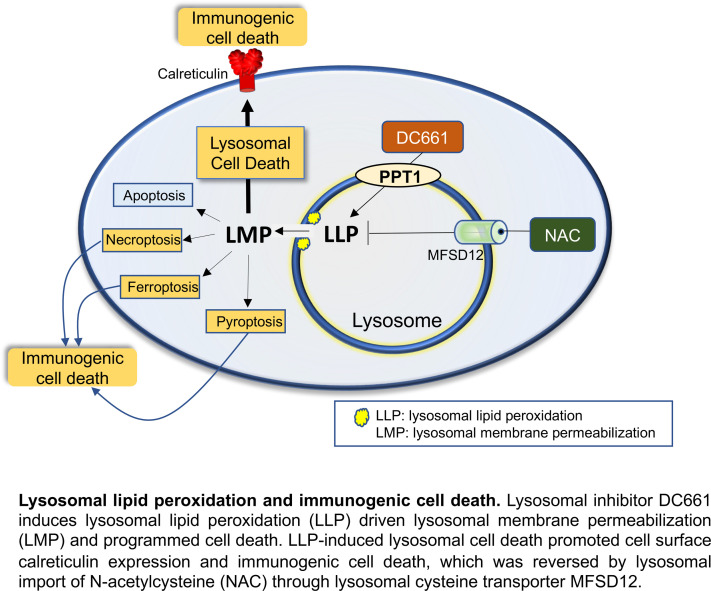

## Introduction

With encouraging activity in clinical trials that involve the lysosomal inhibitor hydroxychloroquine (HCQ) (1), the identification of palmitoyl-protein thioesterase 1 (PPT1) as the molecular target of chloroquine derivatives (2), and the launch of novel PPT1 inhibitors into clinical trials (3, 4), there is a need to understand the mechanism by which lysosomal inhibitors induce cell death and the effect of lysosomal inhibition on tumor immunity. The canonical mechanism of lysosomal cell death described for HCQ involves lysosomal membrane permeabilization (LMP), leakage of cathepsins, and activation of caspase-mediated apoptosis (5, 6). We have previously shown that the lysosomal inhibitor DC661 penetrates cells in the acidic tumor microenvironment and localizes to the lysosome more efficiently than HCQ (2, 7). DC661 produces potent cell death across many cancer cell lines (2). DC661 binds and inhibits PPT1, deacidifies the lysosome, and inhibits autophagy. DC661 also induces LMP and increases levels of cleaved caspase 3 (2, 7). In recent years, novel mechanisms of cell death that have immunogenic consequences have been defined and include necroptosis, ferroptosis, and pyroptosis (8). Lysosome-dependent autophagy has been shown to degrade MHC class I (9), as well as components of the immunoproteasome (10), suggesting that autophagy inhibition can enhance antigen processing. However, the immunogenic effects of lysosomal inhibition and ensuing cell death have not been fully characterized. To address this knowledge gap, we investigated the effects of DC661 on canonical cell death mechanisms to determine if lysosomal cell death is its own form of cell death or simply a precursor to one of the other established mechanisms. We found that HCQ and DC661 induced a significant increase in only a small number of proteins in the melanoma proteome and most of these were autophagy- and apoptosis-related proteins. Inhibitors of programmed cell death (apoptosis, necroptosis, ferroptosis, and pyroptosis) did not mitigate cytotoxic effects after lysosomal impairment by DC661. Lysosomal lipid peroxidation (LLP) is a major driver of LMP-induced cell death associated with calreticulin (CALR) expression on the cell the cell surface. This form of cell death can be reversed only by using the antioxidant N-acetylcysteine (NAC). NAC activity was impaired by inhibition of lysosomal cysteine importer MFSD12. LLP elicited immunogenic phenotypes promoting T cell–mediated killing. These findings demonstrate that lysosomal cell death is a potentially unique form of immunogenic cell death.

## Results

### Lysosomal inhibition induces significant changes in the proteome associated with autophagy and apoptosis.

**Figure 1:**
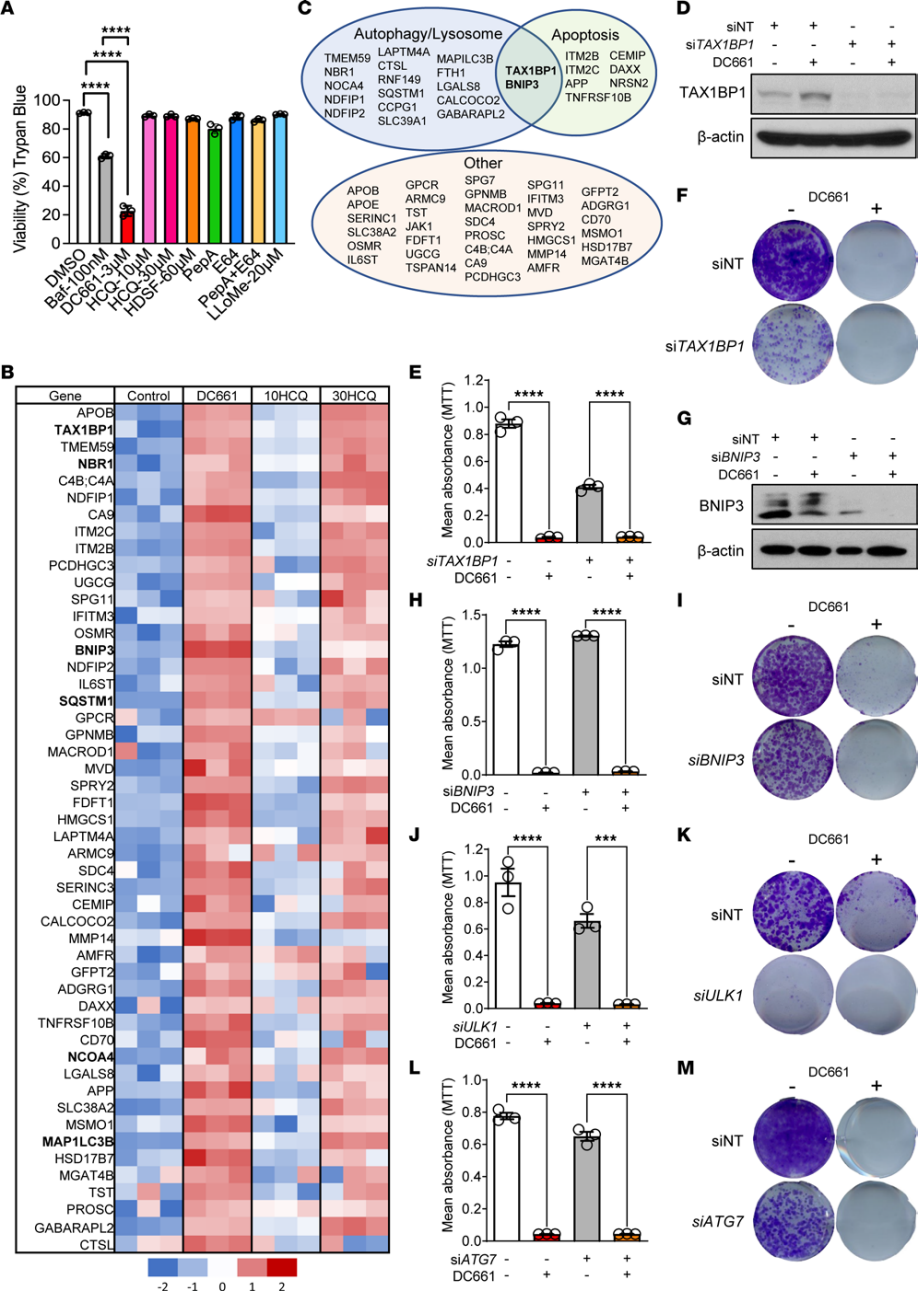
Lysosomal autophagy inhibition induces significant changes in apoptosis and autophagy proteins. (**A**) Trypan blue viability assay of A375P melanoma cells treated with Bafilomycin-A1 (100 nM), DC661 (3 μM), HCQ (10 or 30 μM), hexadecylsulfonyl fluoride (HDSF, 60 μM), pepstatin A (10 μg/mL), E64 (10 μg/mL), or Leu-Leu methyl ester hydrobromide (LLoMe, 20 μM) for 48 hours. (**B**) LC-MS/MS–based proteome analysis of A375P cells treated with DMSO, DC661 (3 μM), or HCQ (10 or 30 μM) for 24 hours. Heatmap of the top 50 elevated proteins in DC661 verses control. Autophagy, apoptosis (names shown in bold), or other signaling pathway proteins significantly elevated (FDR, <5% and fold change, ≥2) in cells treated with 3 μM DC661, 10 μM HCQ, or 30 μM HCQ compared with those treated with vehicle control. (**C**) The autophagy cargo receptor proteins that have proapoptotic effects in cancer cells are shown in a Venn diagram. (**D**–**M**) A375P cells were treated with nontarget siRNA (siNT) or siRNA against *TAX1BP1*, *BNIP3*, *ULK1*, or *ATG7* for 48 hours, followed by treatment with either DMSO or DC661 (3 μM) for 24 hours. (**D** and **G**) Immunoblotting of TAX1BP1 or BNIP3 and β-actin in the whole-cell lysates of A375P cells. (**E** and **H**) Seventy-two-hour MTT assay with 3 μM DC661 or (**F** and **I**) 7-day colony formation assay with 0.3 μM DC661 in A375P cells treated with the indicated siRNA. (**J** and **L**) Seventy-two-hour MTT assay with 3 μM DC661 and (**K** and **M**) 7-day colony formation assay with 0.3 μM DC661 in A375P cells treated with the indicated siRNA. All viability assays were performed in triplicate. ****P ≤ 0.0001. ANOVA test was used when more than 2 groups were compared. See also Supplemental Figure 1 and Supplemental Figure 2, A–C.

To establish the cytotoxic effects of lysosomal inhibitors, we assessed the viability of A375P melanoma, RKO colon carcinoma, and MIA PaCa-2 pancreatic cancer cell lines treated with the vacuolar H+-ATPase inhibitor bafilomycin-A1 (100 nM); PPT1 inhibitors DC661 (3 μM) or HCQ (10 μM or 30 μM); palmitate mimetic hexadecylsulfonyl fluoride (HDSF; 60 μM); cathepsin inhibitors pepstatin A (PepA; 10 μg/mL), E64 (PepA; 10 μg/mL), or PepA+E64; and lysosomal membrane disruptor Leu-Leu methyl ester hydrobromide (20 μM) for 48 hours. Only bafilomycin-A1 and DC661 caused a significant decrease in cell viability across the cancer cell lines (Figure 1A and Supplemental Figure 1A; supplemental material available online with this article; https://doi.org/10.1172/JCI164596DS1), with DC661 producing a more profound reduction in cell viability than bafilomycin-A1. Based on these data and the pharmacological drug–like properties of chloroquine derivatives, we focused our study on chloroquine derivatives as tools to understand lysosomal cell death. An unbiased global proteome analysis was applied to A375P melanoma cells treated with DC661 (3 μM) or the less potent HCQ (10 μM or 30 μM) for 24 hours. Of the 4,264 high-confidence quantified proteins, only 87 and 55 proteins were significantly increased with DC661 (3 μM) and HCQ (30 μM), respectively; additionally, 14 and 15 proteins were significantly decreased with DC661 (3 μM) and HCQ (30 μM), respectively (absolute fold change of greater than 2; *q* < 0.05) with DC661 (3 μM) or HCQ (30 μM), respectively, when compared with vehicle control. The lower dose of HCQ (10 μM) showed no significant protein changes compared with vehicle control. The top 50 proteins that were significantly increased following DC661 treatment were mainly associated with autophagy and apoptosis, and similar changes were observed for the higher dosage of HCQ (Figure 1B). For these reasons, we chose to focus further studies on the more-potent DC661. Examples of some of the largest protein changes associated with autophagy and apoptosis were tax1-binding protein 1 (TAX1BP1; increased 15-fold); BCL2-interacting protein 3 (BNIP3; increased 6-fold); neighbor of BRCA1 gene 1 protein (NBR1; increased 12-fold); sequestosome-1 (SQSTM1/p62; increased 5-fold); nuclear receptor coactivator 4 (NCOA4; increased 3-fold); and LC3B (MAP1LC3B; increased 3-fold). Apolipoprotein B-100 (APOB; increased 66-fold) was derived from fetal calf serum and was not studied further. Immunoblotting confirmed that treatment with DC661 or higher concentrations of HCQ produced marked increases in the expression of autophagy cargo receptors NBR1, TAX1BP1, SQSTM1/p62, and NCOA4 in a dose- and time-dependent manner (Supplemental Figure 1, B and C). CRISPR/Cas9 KO of *PPT1* in A375P cells phenocopied the effects of DC661 on these proteins when compared with their WT counterparts (Supplemental Figure 1D). However, the expression of autophagy cargo receptors and the relative increases induced by DC661 treatment was variable across colon cancer, pancreatic cancer, and other melanoma cell lines in which DC661 demonstrates cytotoxicity (Supplemental Figure 1E). This suggested that autophagy cargo receptors themselves, although increased at the protein level, are unlikely to be responsible for cell death following DC661 treatment. To confirm this, we focused on autophagy cargo receptors TAX1BP1 and BNIP3 because they have known proapoptotic effects in cancer cells (Figure 1C). TAX1BP1 is an autophagy cargo adaptor (11) and also regulates apoptosis induced by protein synthesis inhibitors or DNA-damaging agents in cancer cells (12). Knockdown of *TAX1BP1* by siRNA (Figure 1D) had no effect on DC661-induced cytotoxicity in short-term (Figure 1E) and long-term viability assays (Figure 1F). These results demonstrated that TAX1BP1 does not play an essential role in DC661-mediated cytotoxicity. BNIP3 is a proapoptotic protein that impairs mitochondrial bioenergetics and regulates mitophagy (13). Effective knockdown of *BNIP3* (Figure 1G) had no effect on DC661-induced cytotoxicity (Figures 1, H and I). These results showed that the cytotoxic effects of DC661 are independent of BNIP3 expression. Next, we studied the effects of depleting cells of canonical autophagy genes required for autophagosome production on DC661 efficacy by knocking down unc-51 like autophagy activating kinase 1 (*ULK1*) and autophagy-related gene 7 (*ATG7*). Effective knockdown of *ULK1* or *ATG7* (Supplemental Figure 2, A and B) inhibited autophagic flux (Supplemental Figure 2C) but had no effect on DC661-induced cytotoxicity (Figure 1, J–M). These results showed that the absence of the essential core autophagy machinery does not abrogate the cytotoxic effects of DC661.

### Lysosomal inhibition by DC661 induces multiple programmed cell death pathways.

**Figure 2:**
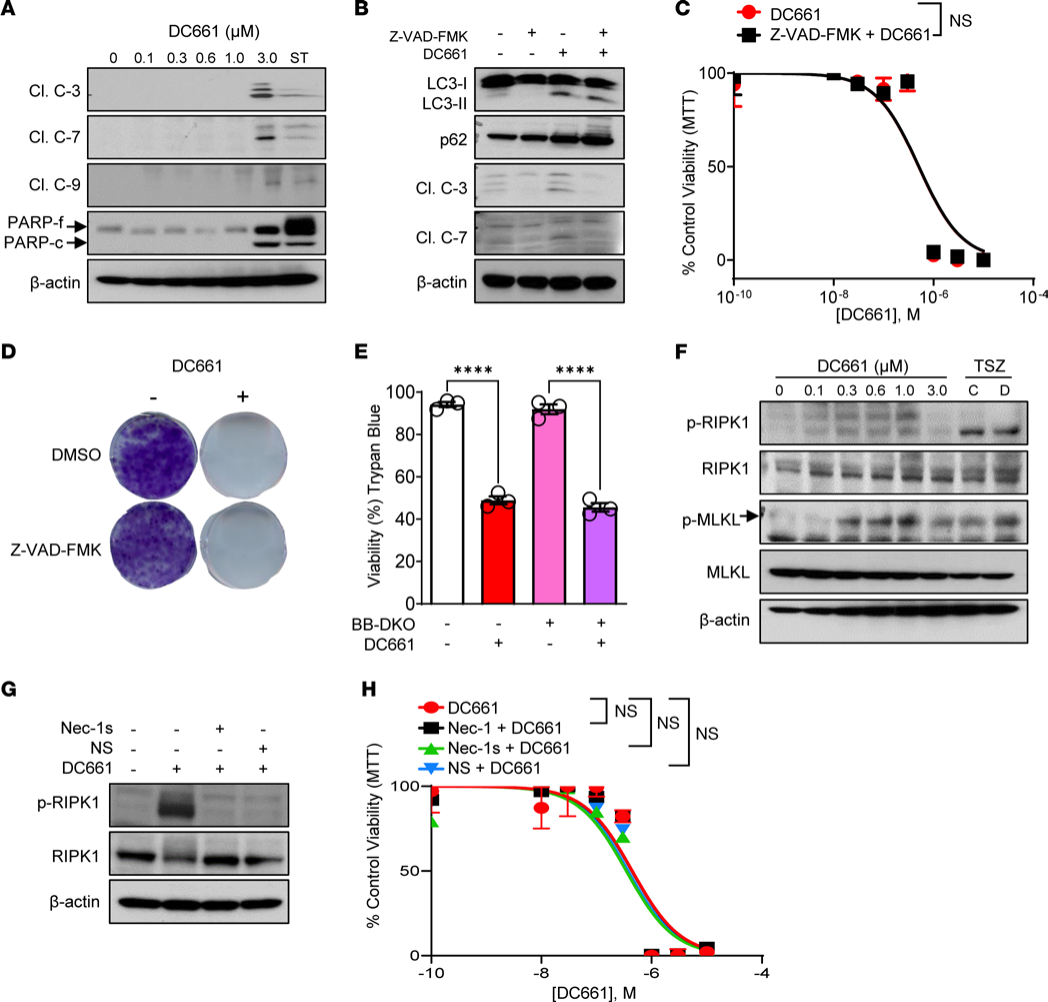
DC661-induced apoptosis and necroptosis. (**A**) Immunoblots of cleaved caspase-3 (Cl. C-3), caspase-7 (Cl. C-7), caspase-9 (Cl. C-9), PARP and β-actin from A375P cell lysates treated with indicated concentrations of DC661 for 24 hours. Staurosporine (ST; 20 ng/mL) was used as a positive control for apoptosis. (**B**) Immunoblots of lysates from A375P cells treated with 3 μM DC661, 80 μM pan-caspase inhibitor Z-VAD-FMK, or both for 24 hours. (**C**) Seventy-two-hour MTT assay plot with increasing concentrations of DC661 (0.01 to 10 μM), with and without Z-VAD-FMK 80 μM. (**D**) Seven-day colony formation assay in A375P cells treated with 0.3 μM DC661, 8 μM Z-VAD-FMK, or their combinations. (**E**) Trypan blue viability assay with and without 3 μM DC661 for 24 hours in FL5.12 and IL-3–dependent *Bax^−/−^Bak^−/−^* (BB-DKO) primary bone marrow cells. (**F**) Immunoblots of RIP1, MLKL, their phosphorylated forms, and β-actin in the lysates of A375P cells treated with DC661 for 24 hours. Necroptosis conventional TSZ (TNF-α, Smac mimetic [SM-164], and Z-VAD-FMK) treatment conditions used included the following: C, pretreatment with Z-VAD-FMK (25 μM, 1 hour), followed by SM-164 (2 μM, 1 hour) and TNF-α (20 ng/mL, 22 hours); D, pretreatment with Z-VAD-FMK (80 μM, 1 hour), followed by SM-164 (100 nM, 1 hour) and TNF-α (20 ng/mL, 22 hours). (**G**) Immunoblots of necroptosis proteins in lysates of A375P cells treated with necroptosis inhibitors necrostatin-1s (Nec-1s, 50 μM) and necrosulfonamide (NS, 2.5 μM) with DC661 1 μM for 24 hours. (**H**) Seventy-two-hour MTT assay plot with DC661 (0.01 to 10 μM), with and without necrostatin-1 (Nec-1, 50 μM), 50 μM Nec-1s, and 2.5 μM NS in A375P cells. All viability assays were performed in triplicate. ****P ≤ 0.0001; ns, nonsignificant. Two-tailed unpaired *t* test between 2 groups (**C**). ANOVA test was used when more than 2 groups were compared (**E** and **H**). See also Supplemental Figure 2, D–G.

The canonical viewpoint is that lysosomal cell death is due to apoptosis (5). Immunoblotting revealed that DC661 treatment resulted in activation of caspase-3, -7, and -9 and cleavage of PARP-1, confirming that DC661 activated apoptosis (Figure 2A). The pan-caspase inhibitor Z-VAD-FMK prevented caspase activation by DC661 but did not inhibit the accumulation of LC3B and p62, demonstrating that apoptosis activation and autophagy blockade are separable following lysosomal inhibition (Figure 2B). Blocking apoptosis with ZVAD-FMK did not enhance or limit DC661 cytotoxicity, suggesting that apoptosis is dispensable for lysosomal cell death (Figure 2, C and D). The cytotoxic effects of DC661 were similar in *Bax/Bak* double-KO primary bone marrow cells incapable of undergoing apoptosis and WT cells (Figure 2E). Blockade of apoptosis had no effect on DC661-induced cytotoxicity in colon cancer and pancreatic cancer cells as well (Supplemental Figure 2, D–F).

Because we found apoptosis to be dispensable for DC661-induced cell death, we investigated whether DC661 induces necroptosis, another form of programmed cell death regulated by receptor-interacting protein kinase (RIPK) and mixed lineage kinase domain-like protein (MLKL). Levels of the phosphorylated and activated forms of RIPK and MLKL were increased following DC661 treatment from 0.1–1 μM (Figure 2F). At 3 μM DC661 there was an absence of phosphorylated RIPK1 but persistent phosphorylated MLKL, suggesting that, at this higher concentration, additional forms of cell death could be engaged. Pretreatment with the RIPK1 inhibitors necrostatin-1 or necrostatin-1s or the MLKL inhibitor necrosulfonamide prevented phosphorylation of RIPK1 after DC661 (1 μM) treatment in melanoma cells (Figure 2G) but failed to rescue DC661-induced cytotoxicity in melanoma cells (Figure 2H) or colon cancer or pancreatic cancer cells (Supplemental Figure 2G). These findings suggest that necroptosis is activated but dispensable for DC661-mediated cell death.

**Figure 3:**
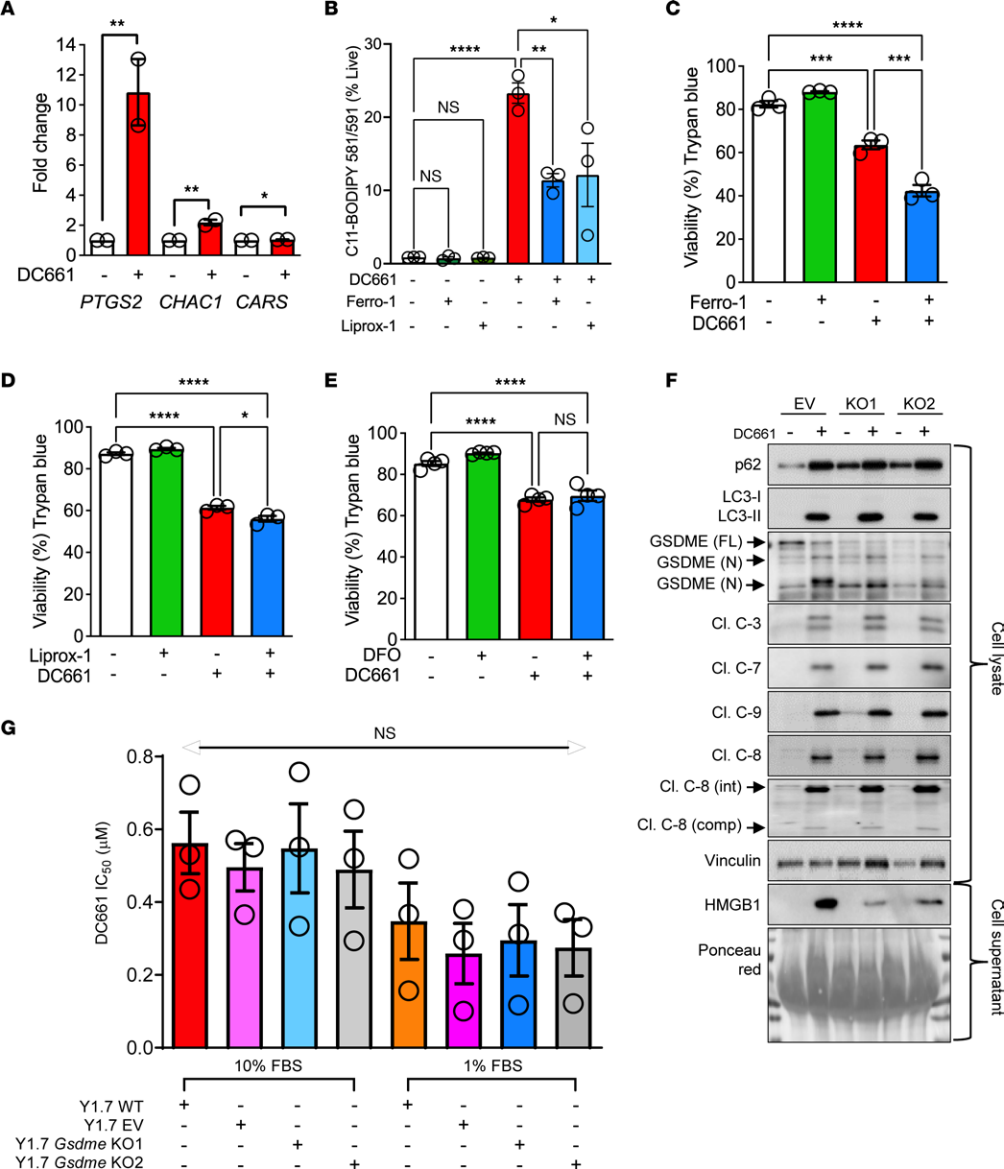
DC661-induced ferroptosis and pyroptosis. (**A**) qRT-PCR showed the fold change increase in the transcriptional expression of *PTGS2*, *CHAC*, and *CARS* in A375P cells treated with 3 μM DC661 for 24 hours. (**B**) A375P cells treated for 24 hours with DC661 (3 μM), liproxstatin-1 (Liprox-1, 2 μM), or ferrostatin-1 (Ferro-1, 10 μM). Lipid peroxidation measured by C-11 BODIPY using flow cytometry. Erastin was used as positive control (see Supplemental Figure 3A). (**C**–**E**) Trypan blue cell viability assay in A375P cells treated with 3 μM DC661, with and without ferroptosis inhibitors (**C**) ferrostatin-1 (Ferro-1, 10 μM), (**D**) liproxstatin-1 (Liprox-1, 2 μM), and (**E**) iron chelator deferoxamine (DFO, 5 μM). (**F**) Western blots were probed for pyroptosis and autophagy proteins in the whole-cell lysates and HMGB1 release in cell supernatant of human WM35 empty vector (EV) and gasdermin-E–KO (KO1 and KO2) cells treated with DC661 1 μM for 48 hours. (**G**) Bar graph showing average DC661 IC_50_ values ± SEM of MTT assays in both 10% and 1% FBS conditions in mouse YUMM1.7 WT, EV, and gasdermin-E–KO (*Gsdme*-KO) cells from 3 independent experiments. Statistical analysis for **I** was applied on ΔCT values. *P ≤ 0.05; **P ≤ 0.01; ***P ≤ 0.001; ****P ≤ 0.0001. Two-tailed unpaired *t* test between 2 groups (**A**). ANOVA test was used when more than 2 groups were compared (**B**–**E** and **G**). See also Supplemental Figures 3–5. All viability assays were performed in triplicate.

Lysosomes are one of the main storage sites for iron. Dysregulated intracellular iron metabolism coupled with decreased reductive capacity can trigger a nonapoptotic cell death known as ferroptosis. A hallmark of ferroptosis is upregulation of prostaglandin-endoperoxide synthase 2 (*PTGS2*), cation transport regulator homolog 1 (*CHAC1*), and cysteinyl-tRNA synthetase (*CARS*) (14). All 3 of these ferroptosis markers were transcriptionally upregulated by DC661 treatment (Figure 3A), suggesting that lysosomal inhibition induces ferroptosis. DC661 induced a fluorescence shift in C11-BODIPY–treated A375P cells, indicating lipid peroxidation, characteristic of ferroptosis. This DC661-induced shift was significantly reversed in the presence of ferroptosis inhibitors ferrostatin-1 or liproxstatin-1 administered at an effective concentration (Figure 3B and Supplemental Figure 3A), further indicating that DC661 induced ferroptosis. However, ferroptosis inhibition did not rescue the cytotoxicity associated with DC661 (Figure 3, C and D). Cotreatment of cancer cells with the iron chelator deferoxamine (DFO) and DC661 did not rescue the cytotoxicity of DC661 (Figure 3E). Treatment with ferrostatin-1, liproxstatin-1, or DFO did not rescue DC661 inhibition of short-term viability or long-term clonogenic growth in melanoma, colon, and pancreatic cancer cells (Supplemental Figure 3, B–E, and Supplemental Figure 4, A–D). These data demonstrate that ferroptosis is activated but dispensable for DC661-mediated cell death.

Pyroptosis is a form of programmed cell death associated with an inflammatory response that involves the activation of caspases that process gasdermin (GSDM), allowing pore formation on the plasma membrane and the subsequent release of damage-associated molecular patterns, such as high-mobility group box 1 (HMGB1). DC661 treatment in multiple melanoma cell lines (A375P, A375, WM35, and WM793) resulted in the activation of initiator caspase-8 and -9 and executioner caspase-7, typical for pyroptosis, and produced cleavage of full-length GSDME, similar in extent to the known pyroptosis inducer PLX4720 and PD0325901 (Supplemental Figure 5A). DC661 produced a stronger extracellular release of HMGB1 than the known pyroptosis inducer, BRAF, and MEK inhibition in 1% FBS (15), reflecting the functional consequence of activated pyroptosis. Next, we tested whether inhibition of pyroptosis by *GSDME* KO would ameliorate the cytotoxic effects of DC661. DC661 treatment induced LC3II and SQSTM1/p62 accumulation and caspase activation, but HMGB1 release was nearly completely abrogated in *GSDME*-KO WM35 human cells, demonstrating that the functional consequence of inhibiting pyroptosis was achieved (Figure 3F). However, DC661 treatment produced equal cytotoxicity in YUMM1.7 WT, empty vector (EV), and *Gsdme* KO1 and KO2 cells in both 10% and 1% FBS conditions (Figure 3G and Supplemental Figure 5B). One common outcome of multiple forms of cell death is the release of LDH. DC661 produced a significant increase in LDH release, and this could not be reversed by apoptosis, necroptosis, or ferroptosis inhibition (Supplemental Figure 5C). These results showed that DC661 induces multiple modes of cell death, including apoptosis and pyroptosis as well as necroptosis and ferroptosis, but none of these modes of cell death are required for DC661-induced cell death.

### Cathepsin inhibition or calcium chelation does not prevent cell death from lysosomal membrane permeabilization.

**Figure 4:**
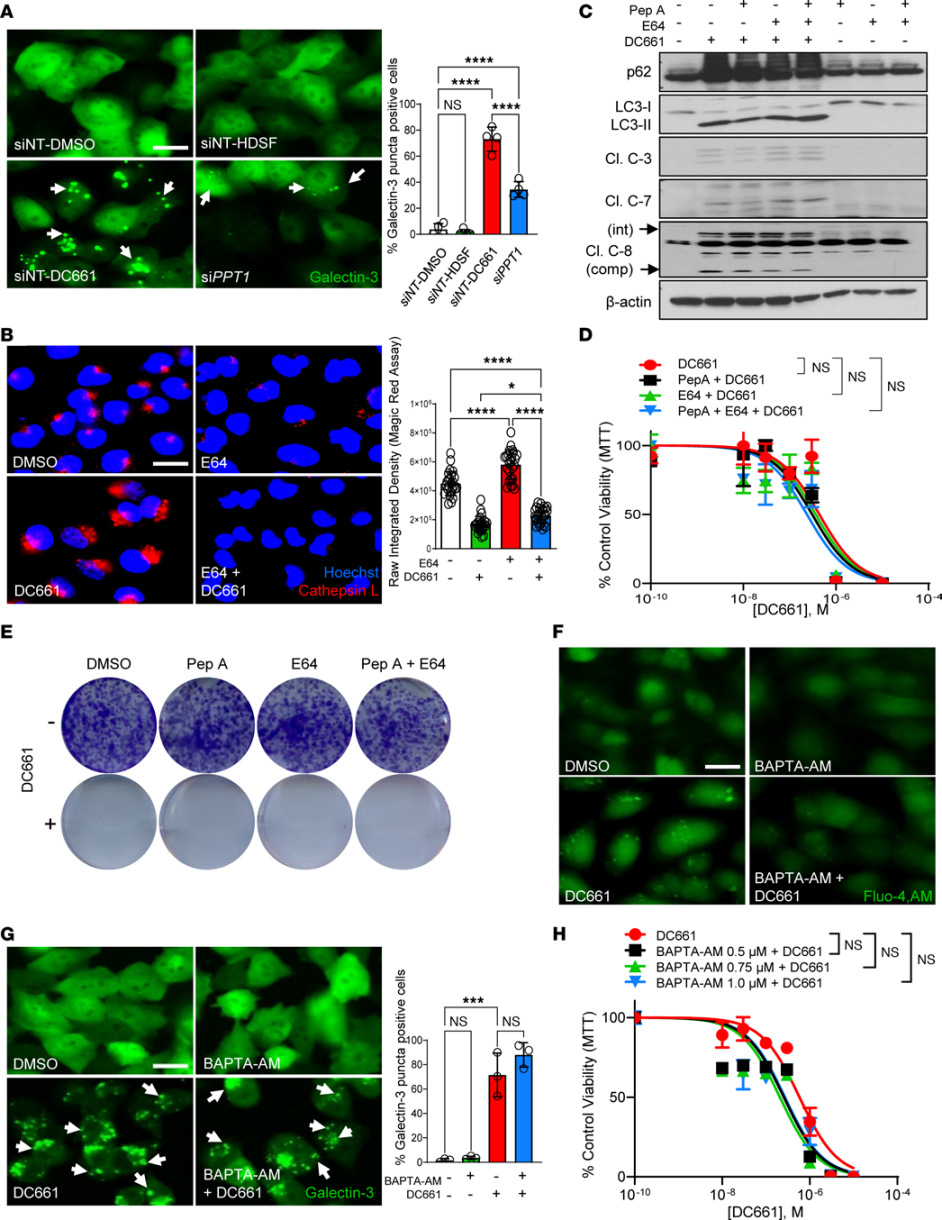
Cathepsin inhibition or calcium chelation does not prevent DC661-induced cell death. (**A**) A375P-galectin-3-EGFP cells were given nontarget siRNA (siNT) or *PPT1* siRNA (si*PPT1*) for 48 hours, followed by treatment with DMSO, 60 μM HDSF, or 3 μM DC661 for 6 hours. (**B**) Cathepsin-L enzyme activity (red) and quantification in A375P cells treated with 3 μM DC661, 10 μg/mL cysteine protease inhibitor E64, or the combination of both for 6 hours. (**C**) Immunoblots for indicated proteins in A375P cell lysates treated with pepstatin A (PepA, 10 μg/mL), 10 μg/mL E64, and PepA+E64 with or without 3 μM DC661 for 24 hours. (**D**) Seventy-two-hour MTT assay plot with increasing concentrations of DC661 (0.01 to 10 μM), with and without PepA, E64, and PepA+E64 in A375P cells. (**E**) Seven-day colony formation assay in A375P cells treated with 10 μg/mL PepA, 10 μg/mL E64, and PepA+E64 with or without 0.3 μM DC661. (**F** and **G**) A375P or A375P-galectin-3-EGFP cells were treated with 3 μM DC661, 1 μM calcium chelator BAPTA-AM, or both for 24 hours. (**F**) Fluorescence images of A375P cells stained with Fluo-4, AM, to detect calcium release. (**G**) A375P-galectin-3-EGFP cells showing galectin-3 puncta (white arrows) and quantification after treatment with DC661, BAPTA-AM, or both. (**H**) Seventy-two-hour MTT assay plot with increasing concentrations of DC661 (0.01 to 10 μM), with and without indicated concentrations of BAPTA-AM in A375P cells. Scale bar: 20 μm. *P ≤ 0.05; ***P ≤ 0.001; ****P ≤ 0.0001. ANOVA test was used when more than 2 groups were compared. All viability experiments were done in triplicate.

Having demonstrated that caspase activation (which is required for apoptosis and pyroptosis) is dispensable for DC661-induced cell death, we hypothesized that cathepsin release from lysosomes could cause caspase-dependent cell death. Chemical (DC661) or genetic (*PPT1* siRNA [*siPPT1*]) inhibition of PPT1 produced lysosomal membrane permeabilization (LMP), while HDSF, a less-potent irreversible inhibitor of PPT1 that gets depleted rapidly in cell culture, could not induce LMP, as measured by galectin-3–positive puncta (Figure 4A). LMP results in the release of cathepsins and other lysosomal contents into the cytoplasm and is thought to be a key proximal feature of lysosome-based cell death. LMP induced by DC661 was associated with a significant increase in cytoplasmic cathepsin-L activity, which was significantly blocked with a cysteine protease inhibitor E64 (Figure 4B). Previous reports have suggested that cathepsin release from fractured lysosomes promotes caspase activation, leading to apoptotic cell death (16–18). Complete cathepsin inhibition did not prevent caspase cleavage (Figure 4C) and did not rescue DC661-induced cytotoxicity in short-term and long-term viability assays in melanoma (Figure 4, D and E), colon cancer, and pancreatic cancer cells (Supplemental Figure 6, A–C). These results counter the canonical view of lysosomal cell death as driven by cathepsin-mediated cell death. Besides cathepsin release from leaky lysosomes, calcium release from lysosomes has been implicated in cellular dysfunction when PPT1 is impaired (19). DC661 treatment resulted in extensive calcium release, which was abrogated by pretreatment of cell-permanent calcium (Ca^2+^) chelator, BAPTA-AM. (Figure 4F). Notably, BAPTA-AM did not prevent DC661-induced LMP (Figure 4G) or caspase cleavage (Supplemental Figure 6, D and E) and, most importantly, did not rescue DC661-induced cytotoxicity (Figure 4H). These findings were also observed in both RKO and MIA PaCa-2 cell lines, in which no significant differences in IC_50_ values were observed with BAPTA-AM and DC661 (Supplemental Figure 6F), which suggested that LMP-associated cell death is not dependent on calcium or cathepsins.

### LLP drives LMP.

**Figure 5:**
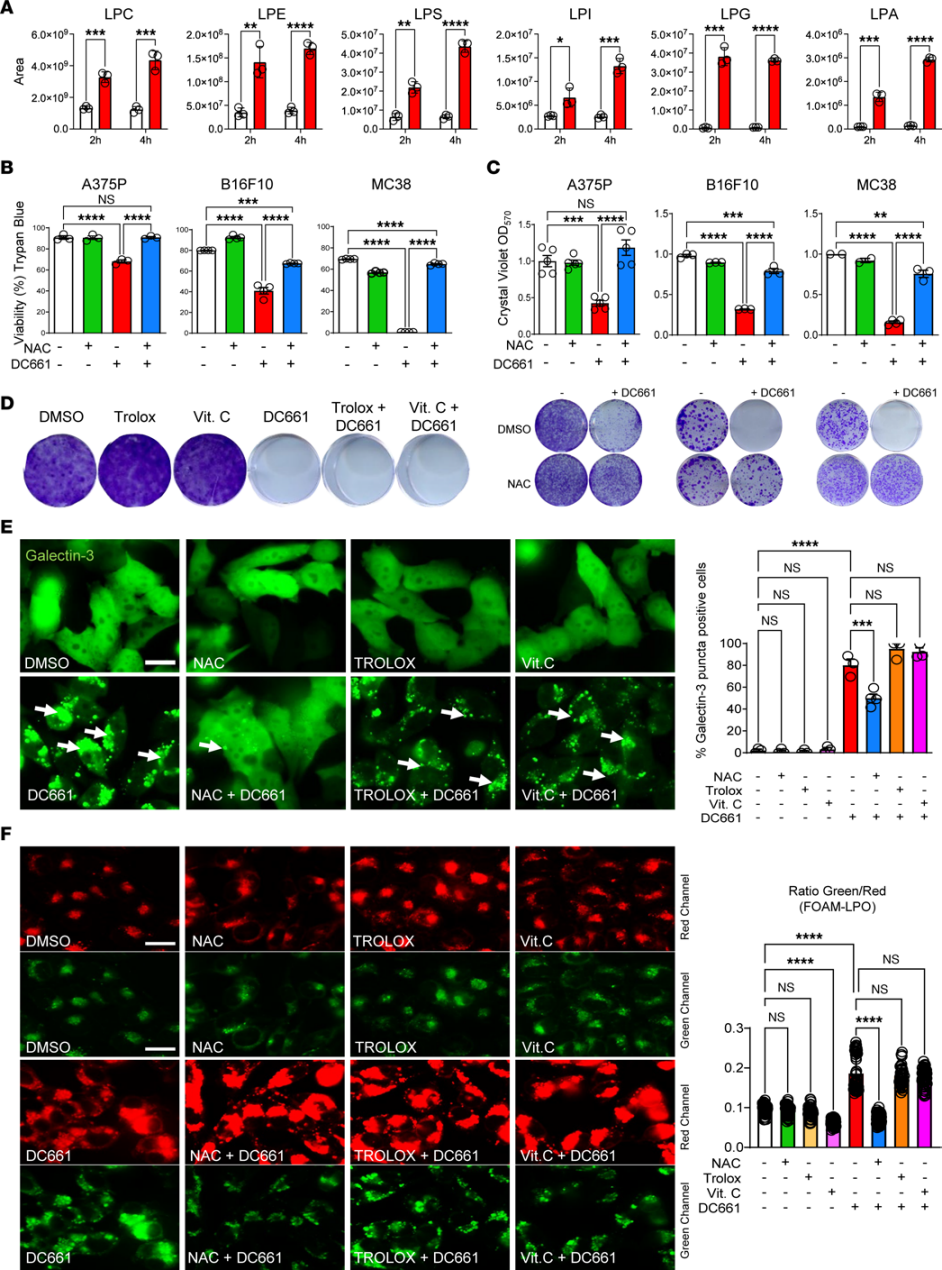
N-acetyl cysteine prevents DC661-induced cell death. (**A**) LC-MS/MS lipidome analysis of A375P cells treated with DMSO (white) or 3 μM DC661 (red) for 2 or 4 hours. Mean ± SD of significantly elevated lysophospholipid classes by unpaired *t* test. LPC, lysophosphatidyl choline; LPE, lysophosphatidyl ethanolamine; LPS, lysophosphatidyl serine; LPI, lysophosphatidyl inositol; LPG, lysophosphatidyl glycerol; LPA, lysophosphatidyl acid. (**B** and **C**) A375P, B16F10, and MC38 cells were treated with 10 mM N-acetyl cysteine (NAC), 3 μM DC661, or both for 72 hours. (**B**) Trypan blue viability assay after 72 hours of treatment. (**C** and **D**) Seven-day colony formation assay in A375P cells treated with 0.1 μM DC661, 1 mM NAC, 100 μM Trolox, and 100 μM vitamin C. (**E** and **F**) A375P-galectin-3-GFP cells or A375P cells were treated with 3 μM DC661, 10 mM NAC, 100 μM Trolox, and 100 μM vitamin C for 24 hours or 6 hours. (**E**) A375P-galectin-3-GFP cells showing galectin-3 (Gal3) puncta (white arrows) and quantification. (**F**) Fluorescence images of A375P cells stained with FOAM-LPO (1 μM, 5 min) to detect LLP. Scale bar: 20 μm. *P ≤ 0.05; **P ≤ 0.01; ***P ≤ 0.001; ****P ≤ 0.0001. ANOVA test was used when more than 2 groups were compared. All viability experiments were done in triplicate.

Having established that inhibitors of major cell death pathways (apoptosis, necroptosis, ferroptosis, and pyroptosis), cathepsins (PepA and E64), and ion chelators (BAPTA-AM [Ca^2+^] and DFO [Fe^2+^]) did not prevent cell death by DC661 and finding evidence of lipid peroxidation by C-11-BODIPY, we performed a global lipidome analysis at 2 and 4 hours after DC661 treatment. DC661 induced early and sustained 3- to 10-fold increases in every class of lysophospholipid (Figure 5A). In contrast, minimal or no changes were seen in phospholipid classes, including phosphatidylcholine, phosphatidylethanolamine, phosphatidylserine, phosphatidylinositol, phosphatidylglycerol, and phosphatidic acid (Supplemental Figure 7A). Lysophospholipid species can be generated by ROS, which oxidize the saturated fatty acid chain that is then cleaved off by phospholipases (20). To determine if antioxidants could prevent ROS-mediated lipid damage, we investigated DC661-induced cytotoxicity in the presence of the pan-ROS scavenger N-acetylcysteine (NAC) (21, 22) and putative lipid peroxidation inhibitors Trolox (23) and vitamin C (ascorbic acid) (24, 25). Unlike any of the other agents tested thus far, cotreatment with NAC rescued DC661-associated cytotoxicity across multiple cell lines (Figure 5B and Supplemental Figure 7B). Long-term CFU assays showed that NAC, unlike all the cell death inhibitors, ion chelators, and cathepsin inhibitors tested herein, prevented the cytotoxic effects of DC661 in A375P, RKO, MIA PaCa-2, B16F10, and MC38 cells (Figure 5C and Supplemental Figure 7C). Interestingly, Trolox and vitamin C did not rescue DC661 cytotoxicity in human melanoma, colorectal cancer, and pancreatic cancer cells and murine melanoma and colorectal cancer cells (Figure 5D and Supplemental Figure 7, D–H). This disparity prompted us to test whether NAC, Trolox, and vitamin C affected LMP. Our results showed that NAC was the only antioxidant that significantly reduced DC661-induced LMP (Figure 5E). We hypothesized that LLP drives LMP production by DC661, which may be reverted by NAC but not by Trolox and vitamin C. To study the process of LLP we used the fluorescent probe FOAM-LPO (26), which specifically localizes to the lysosome and produces a spectral shift from 586 to 512 nm (red to green) when exposed to lipid peroxides. We found that DC661 induced LLP compared with control (Figure 5F). NAC reduced lipid peroxidation in the lysosomes of the DC661-treated melanoma cells, while Trolox and vitamin C failed to reduce LLP (Figure 5F). NAC, Trolox, and vitamin C on their own had no significant effect on lipid peroxidation. Knockdown of *PPT1* (Supplemental Figure 8A), or treatment with high concentrations of HCQ (Supplemental Figure 8B) also induced LMP that could be reversed by NAC, whereas chemical inhibition of ULK1 did not induce LMP (Supplemental Figure 8C). Importantly knockdown of *siPPT1* also included LLP that could be reversed with NAC (Supplemental Figure 8D) These results showed that PPT1 inhibition induces LLP that can be rescued by NAC and is likely the cause of PPT1-inhibition-induced LMP. Further, these findings suggested that LLP is critical for lysosomal cell death.

### Import of cysteine into lysosomes by MFSD12 attenuates lysosomal cell death.

**Figure 6:**
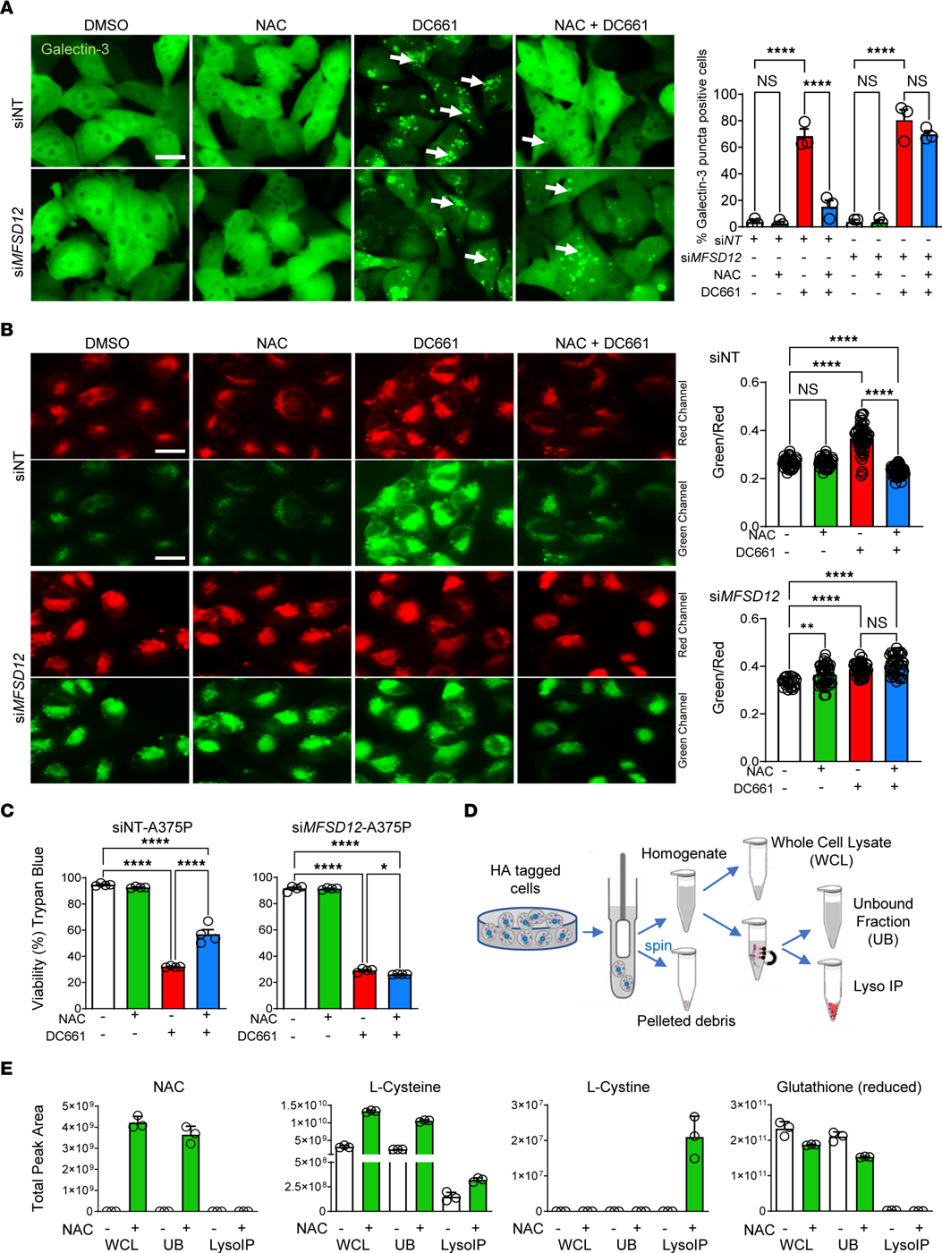
NAC reverses LLP in an MFSD12-dependent manner. (**A**–**C**) A375P-galectin-3-GFP cells or A375P cells were treated with *MFSD12* siRNA or nontarget siRNA (siNT) for 48 hours, followed by treatment with DMSO, 3 μM DC661, or 10 mM NAC for 24 hours, 6 hours, or 72 hours. (**A**) Quantification of galectin-3 puncta in A375P-galectin-3-GFP cells after 24 hours. Galectin-3–positive puncta are shown with white arrows. (**B**) Fluorescence images of A375P cells stained with FOAM-LPO (1 μM, 5 min) to detect LLP after 6 hours. (**C**) Trypan blue cell viability in A375P cells after 72 hours of treatment with DC661, NAC, or both. (**D**) Schematic of lysosomal pull down using Lyso-IP. (**E**) Relative quantification of metabolites in whole-cell lysates (WCL), lysosomal IP unbound fractions (UB), and lysosomal IP bound samples (Lyso IP) with NAC or vehicle treatment after 24 hours. Total peak area accounts for metabolite abundance in the entire sample. Quantifications are depicted as mean ± SD from 3 biological replicates per condition. Scale bar: 20 μm. *P ≤ 0.05; **P ≤ 0.01; ****P ≤ 0.0001. ANOVA test was used when more than 2 groups were compared.

Of the 3 lipid peroxidation inhibitors, only NAC prevented LLP. A recent report showed that major facilitator superfamily domain containing 12 (MFSD12) is an essential component of the cysteine importer for lysosomes and melanosomes (27). We reasoned that NAC, or its metabolite cysteine, is transported into the lysosome through MFSD12, where cysteine gets oxidized to its disulfide, cystine. To test this hypothesis, we compared the ability of NAC to rescue DC661 cytotoxicity in siNT and si*MFSD12* A375P-hGal3-EGFP cells. Our results showed that NAC rescued DC661-induced LMP in siNT cells but did not rescue LMP in si*MFSD12* cells (Figure 6A). NAC reduced LLP of DC661-treated siNT-A375P cells but not si*MFSD12* cells. si*MFSD12* cells treated with DMSO or NAC had increased basal LLP (Figure 6B) compared with siNT cells. While NAC rescued DC661 cytotoxicity in siNT cells, the ability of NAC to rescue DC661 cytotoxicity was completely abrogated in *siMSFD12* cells (Figure 6C). This showed that *MFSD12* knockdown blocks the cytoprotective effects of NAC against DC661. NAC is deacetylated by acylase in the cytosol (28). To determine whether NAC treatment produces an accumulation of cysteine or cystine within lysosomes, we employed the lysosomal immunoprecipitation (Lyso-IP) technique (29) to purify lysosomes from DMSO and NAC-treated A375P cells (Figure 6D and Supplemental Figure 9A) and performed targeted metabolomics to quantify NAC and related metabolites. As expected, NAC was detected in NAC-treated whole-cell lysate and associated unbound fractions. L-Cysteine levels were substantially increased within NAC-treated lysosomes. L-Cystine was only detectable within NAC-treated lysosomes. Strikingly, we did not find a significant increase in glutathione (reduced) in the NAC-treated groups (Figure 6E); this was surprising because it is commonly believed that NAC treatment induces increased production of glutathione, correcting redox balance. These results suggest that cysteine imported into lysosomes through the MFSD12 importer is critical for rescuing LLP, LMP, and lysosomal cell death.

### LLP is immunogenic.

**Figure 7:**
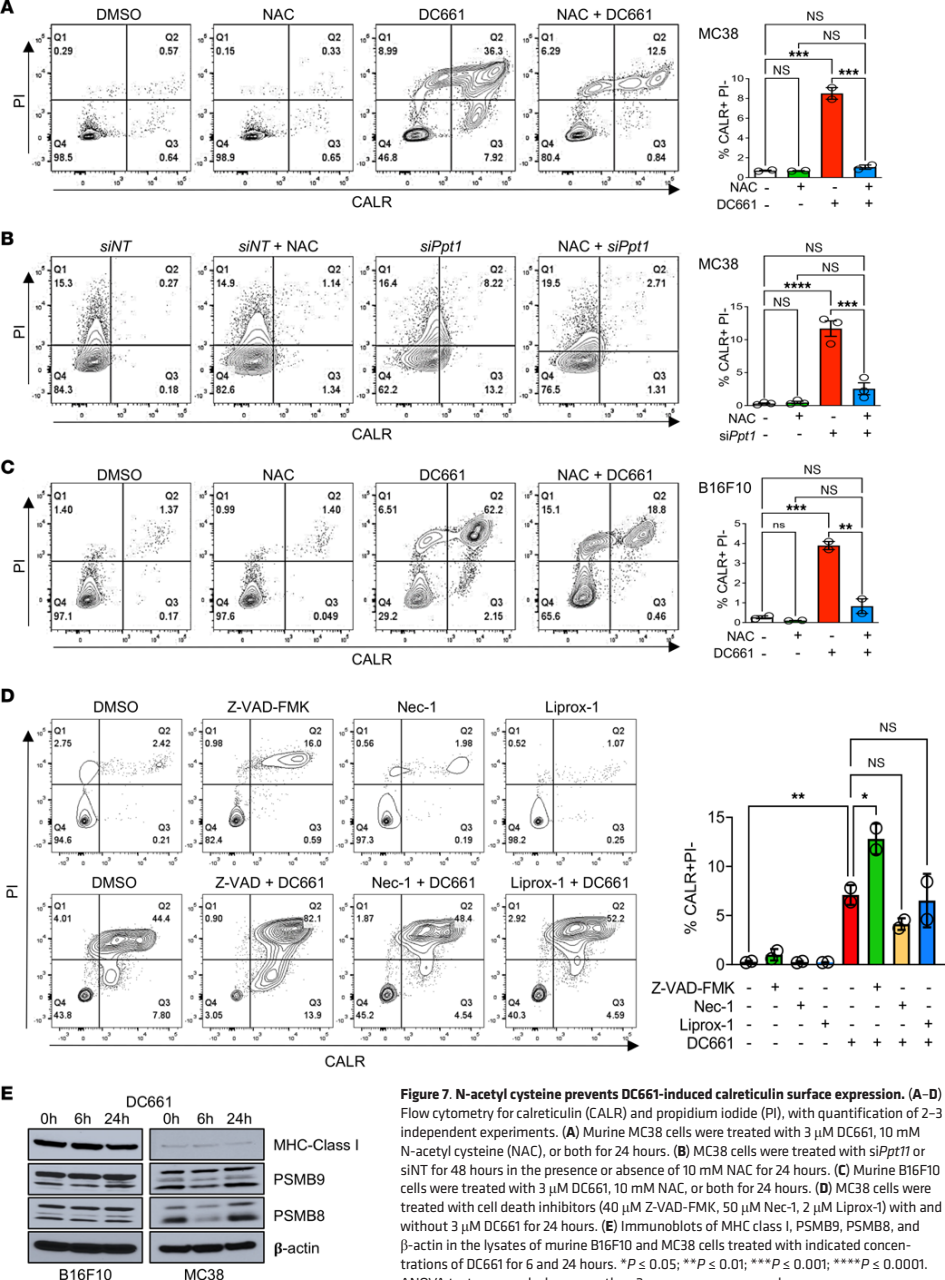
N-acetyl cysteine prevents DC661-induced calreticulin surface expression. (**A**–**D**) Flow cytometry for calreticulin (CALR) and propidium iodide (PI), with quantification of 2–3 independent experiments. (**A**) Murine MC38 cells were treated with 3 μM DC661, 10 mM N-acetyl cysteine (NAC), or both for 24 hours. (**B**) MC38 cells were treated with si*Ppt11* or siNT for 48 hours in the presence or absence of 10 mM NAC for 24 hours. (**C**) Murine B16F10 cells were treated with 3 μM DC661, 10 mM NAC, or both for 24 hours. (**D**) MC38 cells were treated with cell death inhibitors (40 μM Z-VAD-FMK, 50 μM Nec-1, 2 μM Liprox-1) with and without 3 μM DC661 for 24 hours. (**E**) Immunoblots of MHC class I, PSMB9, PSMB8, and β-actin in the lysates of murine B16F10 and MC38 cells treated with indicated concentrations of DC661 for 6 and 24 hours. *P ≤ 0.05; **P ≤ 0.01; ***P ≤ 0.001; ****P ≤ 0.0001. ANOVA test was used when more than 2 groups were compared.

Some forms of regulated cell death induced by DC661 (pyroptosis, necroptosis, ferroptosis) have been identified as immunogenic. Previously, immunogenic cell death has been described for chemotherapy drugs based on characteristic features, which include display of damage-associated molecular patterns, including cell surface expression of CALR and release of HMGB1 protein and adenosine triphosphate (ATP) (30). CALR acts as an “eat-me” signal when exposed on cell surfaces during cell stress. Extracellular release of HMGB1 and ATP acts as a “find-me” signal recognized by phagocytic cells. We compared two models: B16F10 cells, which in syngeneic tumor models are almost completely devoid of tumor-infiltrating lymphocytes, and MC38 cells, which in syngeneic tumor models do have tumor-infiltrating lymphocytes. First, we demonstrated that DC661 or si*Ppt1* treatment significantly induces surface CALR expression that can be completely abrogated with NAC cotreatment in MC38 cells (Figures 7, A and B). Similar findings were observed in B16F10 cells (Figure 7C). Inhibitors of major cell death pathways (apoptosis, necroptosis, ferroptosis, and pyroptosis) failed to prevent the surface expression of CALR (Figure 7D). To determine if immunogenic cell death induced by DC661 is due to MHC class I upregulation, B16F10 and MC38 cells were treated with DC661 (3 μM) or DMSO for 24 hours. We found that DC661 treatment did not increase the expression of MHC class I and immunoproteasome upregulation (Figure 7E and Supplemental Figure 9, B and C).

**Figure 8:**
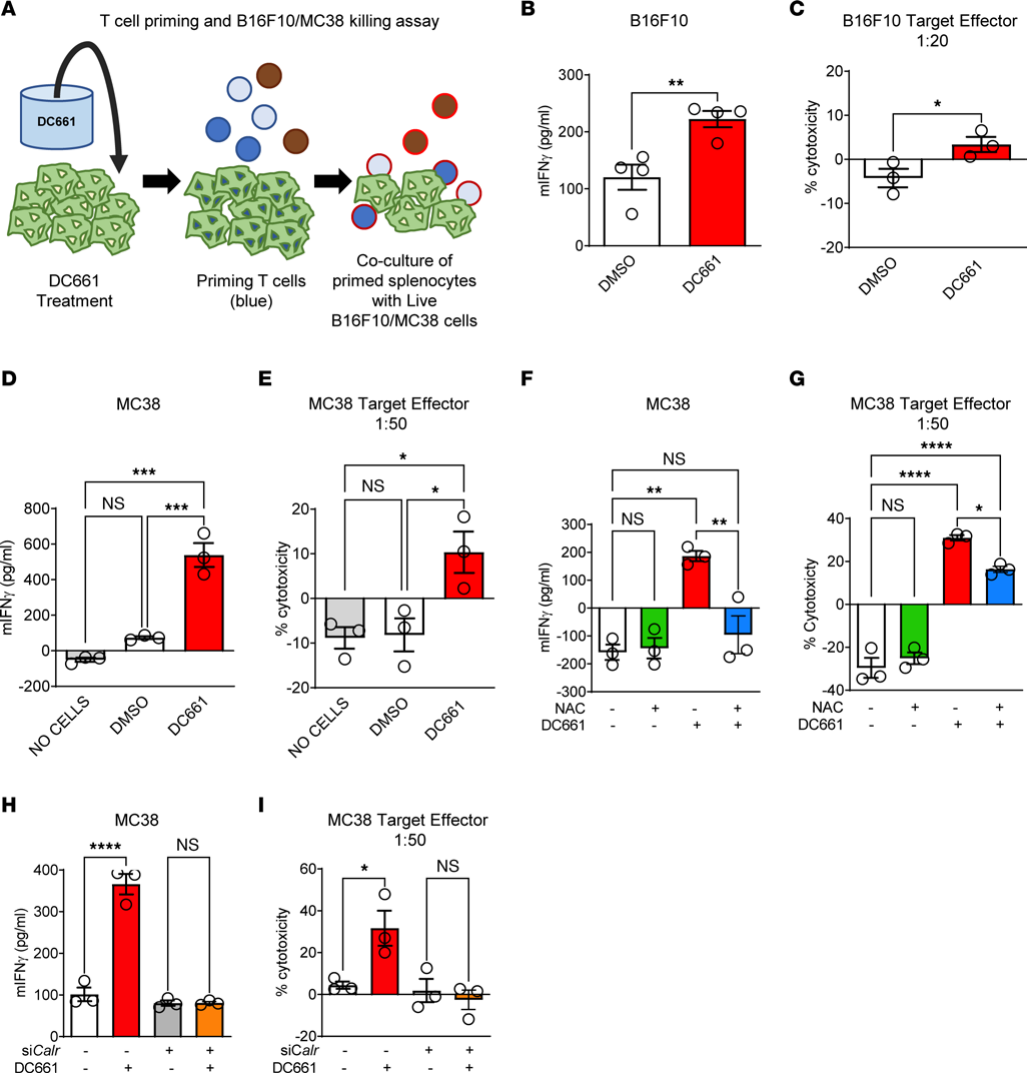
DC661-induced calreticulin surface expression primes T cells against tumor cells. (**A**) Schema of experimental setup of splenocyte priming and coculture experiments to measure cytotoxicity in vitro by DMSO or DC661 treatment for 24 hours. (**B** and **D**) Measurement of splenocyte-secreted IFN-γ upon coculturing syngeneic splenocytes with B16F10 or MC38 cells treated with DMSO or DC661. (**C** and **E**) Measurement of percentage cytotoxicity (LDH release assay) of B16F10 or MC38 cells by DMSO- or DC661-primed splenocytes. (**F** and **G**) MC38 cells were treated with 3 μM DC661, 10 mM NAC, or both for 24 hours and then used to prime splenocytes that were used for the cytotoxicity assay. IFN-γ and percentage cytotoxicity are shown. (**H** and **I**) For calreticulin (*Calr*) genetic inhibition, MC38 cells were treated with *calreticulin*/*calregulin* (*Calr*) siRNA or nontarget siRNA (siNT) for 48 hours, followed by treatment with either DMSO or 3 μM DC661 for 24 hours. These cells were then applied to the T cell priming and cytotoxicity assay. Measurement of IFN-γ and percentage cytotoxicity are shown. All experiments were done in triplicate. *P ≤ 0.05; **P ≤ 0.01; ***P ≤ 0.001; ****P ≤ 0.0001. ANOVA test was used when more than 2 groups were compared.

To understand the effects of autophagy inhibition on T cell priming, we performed an in vitro priming and coculture experiment using C57BL6/J splenocytes as previously described (31). For priming, we exposed splenocytes to DC661- or DMSO-treated B16F10 or MC38 cells. Next, these primed splenocytes were cultured with live B16F10 or MC38 cells, and cytotoxicity was measured (Figure 8A). Splenocytes primed with DC661-treated B16F10 cells produced a significant increase in IFN-γ compared with splenocytes exposed to DMSO-treated B16F10 cells. Primed T cell–mediated killing of proliferating B16F10 cells was significantly increased in DC661-primed splenocytes when compared with DMSO-primed splenocytes (Figure 8, B and C). MC38 cells treated with DC661 produced even more IFN-γ and primed T cell cytotoxicity when compared with B16F10 cells (Figure 8, D and E). NAC cotreatment with DC661 was able to completely abrogate IFN-γ release from splenocytes and blunt primed T cell cytotoxicity (Figure 8, F and G). Knockdown of calreticulin by *siCalr* in MC38 cells (Supplemental Figure 9D) abrogated the priming efficacy of DC661 treatment and significantly reduced primed T cell cytotoxicity (Figure 8, H and I). Taken together, these data support a mechanistic role of LLP-associated CALR upregulation in promoting antitumor cell T cell immunity.

**Figure 9:**
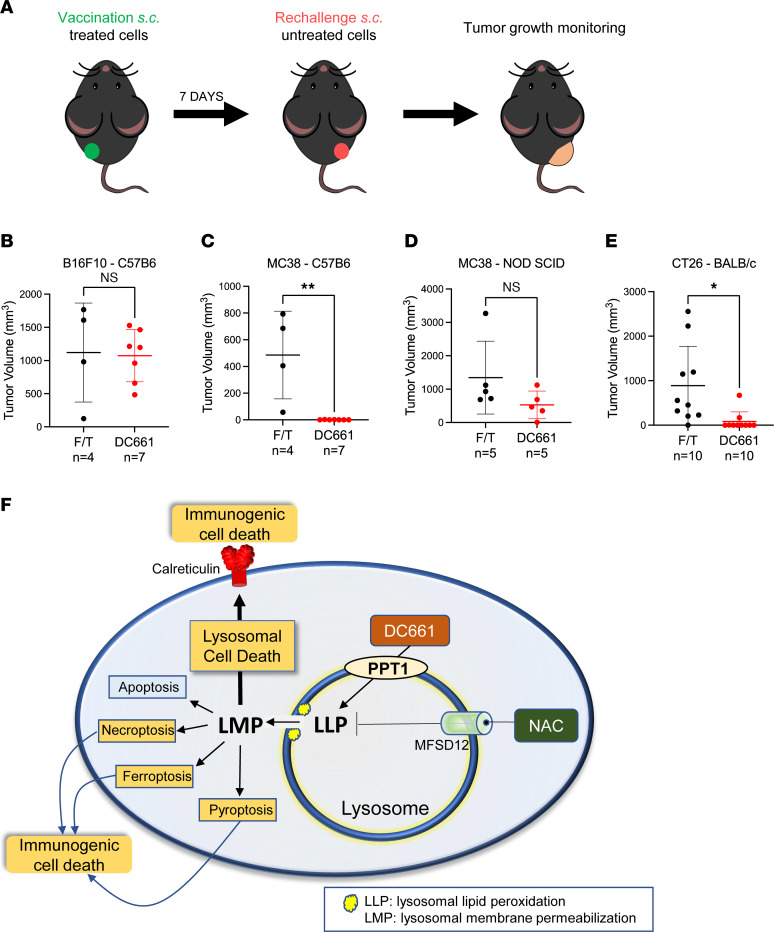
Inoculation of DC661-treated cells produces tumor rejection in specific contexts. (**A**) Schema of tumor vaccination model. (**B**–**E**) Cells were treated with DMSO or DC661 for 36 hours and then s.c. injected (1.8 × 10^5^ B16F10 cells, 1.5 × 10^6^ MC38 cells, or 3.0 × 10^6^ CT26 cells per mouse) into the left flank of immunocompetent syngeneic C57BL/6J, BALB/c mice, or immunodeficient NOD/SCID mice. Freeze-thawed (F/T) DMSO-treated cells were used as control. One week later, all mice were rechallenged and s.c. injected with live untreated cells (3 × 10^4^ B16F10 cells, 2 × 10^5^ MC38 cells, or 5 × 10^5^ CT26 cells per mouse) into the right flank of corresponding vaccinated mice. Dot plot of final tumor volumes for each individual mouse in each treatment group shown. *n* = 4–10 per group. (**F**) Illustration of DC661-induced lysosomal lipid peroxidation and immunogenic cell death.

Next, we expanded these in vitro findings to an in vivo antitumor vaccination study in immunocompetent and immunodeficient mice models. For this assay, in vitro freeze-thawed or DC661-treated B16F10 or MC38 cells were injected s.c. on the left flank to protect immunocompetent C57BL/6 or NOD/SCID mice against rechallenge with live tumor cells of the same kind injected 7 days later into the right flank (Figure 9A). DC661-treated B16F10 cells failed to prevent tumor rejection despite in vitro assays, suggesting that DC661 induced immunogenic cell death (Figure 9B and Supplemental Figure 9E). In contrast, inoculation of one flank with DC661-treated MC38 cells promoted complete rejection of live MC38 cells implanted on the opposite flank (Figure 9C and Supplemental Figure 9F). To determine if adaptive immunity was critical for the vaccine effect DC661 seemed to have with MC38 tumors, we repeated the experiment in NOD/SCID mice. Strikingly, we found that DC661-treated MC38 cells did not induce rejection of rechallenge tumors in immunodeficient NOD/SCID mice (Figure 9D and Supplemental Figure 9G), indicating that adaptive immunity was required for the observed vaccine effect. To test the robustness of this finding, we selected another well-known immunogenic mouse cancer cell line, CT26, and performed the vaccination experiment as above. As expected, implantation of DC661-treated CT26 cells in one flank of the mouse produced a vaccine-like effect and prevented the outgrowth of live CT26 cells implanted on the other flank (Figure 9E and Supplemental Figure 9H). Our findings suggested that DC661-induced LLP is critical for LMP-mediated immunogenic cell death, which is reversed by the import of cysteine into lysosome by lysosomal transporter MFSD12 (Figure 9F).

## Discussion

Lysosomal inhibition appears to be a promising therapeutic approach in preclinical studies, and HCQ clinical trials have produced encouraging but mixed results (1, 32). PPT1 is the molecular target of HCQ, and more-potent PPT1 inhibitors, such as DC661 (2) and GNS561 (3), induce LMP-mediated cell death in vitro. The precise mechanism of lysosomal cell death and its role in tumor immunogenicity have not been fully elucidated.

Antitumor immunity is enhanced when autophagy inhibition is combined with immunotherapy (9, 10, 31). Proposed mechanisms for cell-intrinsic immunogenicity following autophagy inhibition include MHC class I and immunoproteasome upregulation, which both support improved antigen processing and presentation. In addition, loss of autophagy proteins or autophagy inhibition by chloroquine augmented CD8^+^ T cell response by increasing surface levels of MHC class I in dendritic cells (33). We previously showed that systemic PPT1 inhibition can repolarize macrophages from an M2 to M1 phenotype. PPT1 inhibitors can enhance STING levels, leading to IFN release and augmentation of T cell–mediated killing in melanoma models (31). Here, we demonstrated that LLP itself produces a tumor cell–intrinsic immunogenic form of cell death.

We found that lysosomal inhibition induced very few protein changes in cancer cells, and the most significantly elevated proteins included autophagy cargo receptors, apoptosis regulators. Our approach targeting some of these genes across functions demonstrated that drug-induced proteins were not likely the main regulators of cell death. Genetic inhibition of *ULK1* or *ATG7* also did not rescue DC661 cytotoxicity. We demonstrated that lysosomal inhibition activates multiple forms of programmed cell death, including apoptosis, necroptosis, ferroptosis, and pyroptosis, but each of these was dispensable for drug-induced cytotoxicity. Of note, programmed cell death mechanisms can overlap and cotargeting of multiple cell death mechanisms could provide cytoprotection against cell death following lysosomal membrane permeabilization. Our work has ruled out cathepsin- and calcium-dependent mechanisms for lysosomal cell death and highlighted the importance of LLP as the critical determinant of cell death. We found evidence of LLP that was reversible by an antioxidant that was transported into the lysosome, NAC, and was critical for lysosomal membrane permeabilization and cytotoxicity. NAC was the only agent able to mitigate or reverse DC661-induced cell death, and this capacity was dependent on the presence of the lysosomal cysteine transporter MFSD12. A lack of lysosomal penetration is likely why other putative lipid peroxidation inhibitors, Trolox and vitamin C, were unable to prevent DC661 cytotoxicity. NAC is converted to cysteine, which is imported into the lysosomes and oxidizes to its disulfide form, cystine. Cystine is exported to the cytosol by another lysosomal transporter, cystinosin, where it is reduced to cysteine that remobilizes internal nutrient sources, reactivates target of rapamycin complex 1, and promotes autophagy (34). This oxidation-reduction cycling of cysteine to cystine (lysosomes) and back to cysteine (cytosol) could be a possible rescue mechanism of NAC against DC661 cytotoxicity or more general lysosomal injury across other disease contexts where NAC has proven to be useful therapeutically (35). NAC not only reversed DC661-induced LLP and LMP, but also surface expression of the immunogenic cell death marker calreticulin. Cell surface expression of CALR protein was required for the enhanced T cell–mediated cytotoxicity induced by DC661-primed splenocytes, demonstrating that lysosomal inhibition produces a specific form of cell-intrinsic immunogenicity.

While previous studies have demonstrated that lysosomal inhibition can enhance the antitumor activity of immune checkpoint inhibition in established flank tumors (9, 31), our study is the first to our knowledge to demonstrate a vaccine-like effect for MC38 tumors but not B16 tumors in tumor cells pretreated with DC661 prior to implantation. This demonstrates that lysosomal cell death can induce cell-intrinsic immunogenicity, but these changes by themselves are not likely enough to reverse an “immune cold” tumor microenvironment into an “immune hot” tumor microenvironment. Our previous studies in “immune cold” tumor microenvironment models B16 and the *BRaf^CA^ Pten^loxP^ Tyr:CreER^T2^* genetically engineered mouse models (31) demonstrated that systemic lysosomal inhibition produced effects on tumor-associated macrophages and myeloid cell–derived suppressor cells that were sufficient to enhance the efficacy of immunotherapy. It may be the case that cell-intrinsic immunogenicity also plays a role in these immune cold tumors that become more responsive to ICD following lysosomal inhibition. The vaccine-like effect of lysosomal inhibition observed in a controlled laboratory environment may or may not translate into the clinic, but it could explain why some patients treated with dabrafenib, trametinib, and HCQ in previously treated *BRAF* mutant melanoma had such a deep and durable response this regimen (32).

Further study is needed to understand how PPT1 inhibition produces lysosomal ROS and lipid peroxidation. PPT1-dependent regulation of the V-ATPase and lysosomal acidification is not a sufficient explanation because bafilomycin inhibits lysosomal acidification but does not produce LMP (data not shown). The implications of these findings suggest that lysosomal inhibitors that enhance tumor cell–intrinsic immunogenicity can be rationally combined with therapies that enhance T cell activation, or infiltration into the tumor microenvironment, potentially yielding synergistic effects.

## Methods

### Cell culture.

Human A375P (CRL-3224), RKO (CRL-2577), DLD-1 (CCL-221), MIA PaCa-2 (CRL-1420), A549 (CRM-CCL-185), and mouse B16F10 (CRL-6475) cell lines were purchased from ATCC. Human A375 (CRL-1619, ATCC), WM35, WM793, and mouse YUMM1.7 (WT, *Gsdme* EV, and *Gsdme* KO1 and KO2) lines were obtained in-house. Mouse MC38 and CT26 cells were provided by Andy Minn, University of Pennsylvania. FL5.12 and IL-3–dependent *Bax^−/−^Bak^−/−^* (*Bax/Bak* DKO) primary bone marrow cells were obtained from Kathryn E. Wellen, University of Pennsylvania. The human Panc-1 (CRL-1469, ATCC) cell line was provided by Ben Z. Stanger, University of Pennsylvania. The mouse YUMMER 1.7 cell line was obtained from Xiaowei (George) Xu, University of Pennsylvania. All cell lines were tested for Mycoplasma biannually by University of Pennsylvania Core facilities and authenticated using short-tandem repeat fingerprinting by Wistar Institute Genomics core. A375P, RKO, DLD-1, A549, CT26, FL5.12, and *Bax/Bak* DKO cell lines were cultured in RPMI 1640 (Invitrogen, 11875); A375, MIA PaCa-2, Panc-1, B16F10, and MC38 cell lines were cultured in DMEM (Invitrogen, 11995); and YUMMER1.7, YUMM1.7 WT, *Gsdme* EV, and *Gsdme* KO1 and KO2) cells (15) were cultured in DMEM/F12 50/50 (Corning, 10-092-CV). Culture media were supplemented with 10% fetal bovine serum (12306C, MilliporeSigma) and 1× antibiotic antimycotic solution (Gibco, 15140-122). FL5.12 and *Bax/Bak* DKO cell lines were maintained in complete RPMI media supplemented with 50 μM β-mercaptoethanol (Life Technologies, 21985-023), 10 mM HEPES (H3537, MilliporeSigma), and 0.35 ng/mL and 3.5 ng/mL IL-3, respectively. YUMMER1.7 and YUMM1.7 (WT, *Gsdme* EV, and *Gsdme* KO) cell lines were maintained in complete media supplemented with 1× MEM NEAA (Gibco, 11140-050). WM35 and WM793 cells were cultured in MCDB media 153 (MilliporeSigma, M7403), containing 10% FBS in 1× Leibovitz L-15 medium (Corning, 10-045-CV), 7.5% w/v sodium bicarbonate (Corning, 25-035-CI), 1× antibiotic antimycotic solution (Gibco, 15140-122), and 5 μg/mL insulin (MilliporeSigma, I0516). Cells were grown at 37°C in the presence of 5% CO_2_.

### Chemicals and reagents.

Chemicals purchased included HCQ Sulfate (Spectrum Chemicals, 747-36-4) and DC661 (Selleckchem, S8808). Fluo-4, AM (Thermo Fisher Scientific, F14201) was used to stain the cells for calcium as per the manufacturer’s instructions. A list of antibodies and inhibitors is provided in Supplemental Table 1 and Supplemental Table 2.

### Protein extraction and digestion for liquid chromatography–tandem mass spectrometry analysis for proteomics.

0.7 × 10^6^ A375P melanoma cells were cultured in 60 mm culture dishes. Cells were treated with DMSO (control), DC661 (3 μM), HCQ (10 μM), or HCQ (30 μM) for 24 hours at approximately 50% confluence. Frozen cell pellets were lysed with 50 mM Tris pH 7.4, 1% SDS, 150 mM NaCl, 1 mM EDTA, 0.15 mM PMSF, 1 μg/mL pepstatin, and 1 μg/mL leupeptin. Clarified lysates (10 μg each) were electrophoresed 0.5 cm into a bis-tris gel followed by fixing and staining with colloidal Coomassie. Each 0.5 cm gel lane was excised, destained, reduced with tris (2-carboxyethyl) phosphine, alkylated with iodoacetamide, and digested with trypsin as described previously (36). Digests (1 μg) were analyzed by liquid chromatography–tandem mass spectrometry (LC-MS/MS) on a Q-Exactive Plus mass spectrometer (Thermo Fisher Scientific) in-line with a nanoAQUITY UPLC (Waters). Analytical separation was performed on a 1.7 μm × 250 mm Peptide BEH C18 column (Waters, 186003546) using a 245-minute gradient with 0.1% formic acid in water (mobile phase A) and acetonitrile (mobile phase B) as follows: 5%–30% B over 225 minutes, 30%–80% B over 5 minutes, 15-minute hold at 80% B, and return to initial conditions. Full MS spectra were acquired at 70,000 resolution, with a scan range of 400–2,000 *m/z*, automatic gain control target of 3 × 10^6^ ions, and maximum injection time of 50 milliseconds. Data-dependent MS2 spectra were acquired for the top 20 most abundant ions at 17,500 resolution, with an isolation width of 1.5 *m/z*, automatic gain control target of 5 × 10^4^ ions, and maximum injection time of 50 milliseconds. Peptide match was set to preferred, and unassigned and singly charged ions were rejected (37).

Raw MS data were analyzed using MaxQuant 1.6.5.0 (https://maxquant.net/perseus/) with a Uniprot human sequence database (accessed on October 10, 2019) and a common contaminants database, including trypsin, keratins, bovine proteins, and mycoplasma (38). Tryptic peptide specificity with a maximum of 2 missed cleavages, fixed modification on cysteine (carbamidomethylation), and variable methionine oxidation or N-terminal acetylation were used in the search (39). A cutoff of 1% FDR was used for peptides and proteins. Match between runs was enabled; proteins were quantified using label-free quantitation (40).

Statistical analysis was performed using Perseus 1.6.2.3 (https://maxquant.net/perseus/) (41, 42). Proteins were required to be identified by at least 3 unique peptides and have 3 valid values (non-zero quantitation) within a sample group, and contaminants and reverse proteins were filtered from the data set. Missing values were imputed from a normal distribution. Pairwise comparisons between conditions were performed at the protein level using 2-tailed Student’s *t* test with permutation-based FDR with s0 = 0.1 and 250 randomizations. Significant changes were defined as FDR of less than 5% and an absolute fold change greater than 1.5 or 2.0, as specified.

### Lyso-IP.

A375P cells were infected with pLJC5-Tmem192-3xHA lentivirus and selected using 1 mg/mL puromycin (MilliporeSigma, P4512). Approximately 3 × 10^6^ HA tagged A375P cells were treated with either 10 mM NAC or water vehicle control for 24 hours in culture conditions previously described. After treatment, cells were washed in PBS, harvested in 0.5 mL cold KPBS, and gently homogenized using 20 strokes in a 2 mL Dounce homogenizer. About 2.5% of the homogenate was reserved for whole-cell lysate analysis, and the remainder was centrifuged at 3,000*g* for 2 minutes at 4°C to remove cell membrane debris. Homogenate supernatant was then transferred to a clean 1.5 mL tube and incubated with 50 μL anti-HA beads (Pierce, 88836) or anti-DDK/Flag beads (OriGene, TA150042) for 15 minutes at 4°C. Samples were then precipitated by placing tubes on DynaMag (Thermo Fisher Scientific, 12321D) and rocked gently for 2 minutes at room temperature. Supernatant was reserved for unbound fraction analysis. IP was washed 3 times with KPBS containing 8 mM CaCl_2_. The Lyso-IP was extracted in 80% MeOH for metabolomics analysis.

### Lipid extraction and analysis using LC-MS/MS for global lipidome.

Melanoma A375P cells were seeded in 60 mm dishes at 0.7 × 10^6^ cells per dish. When cells were at approximately 50% confluence, they were treated with DMSO (control), DC661 (3 μM), or HCQ (30 μM) for 24 hours. Cells were washed 2 times with HBSS, scraped into ice-cold methanol, and transferred to glass tubes for lipid extraction with chloroform/methanol/0.88% NaCl (2:1:1) containing an EquiSPLASH internal lipid standard (Avanti Polar Lipids). Samples were vortexed and then water bath sonicated for 5 minutes on ice. After centrifugation at 500*g* for 15 minutes at 40°C, the lower phase was transferred to another glass tube. The upper phase was reextracted using a synthetic lower phase. After sonication and centrifugation, the lower phase was combined with the first lower phase collection. The samples were dried under nitrogen, resuspended in 10% chloroform/90% methanol, and transferred to glass LTQ vials.

Lipid samples were analyzed on a Thermo Fisher Scientific Q-Exactive HF-X mass spectrometer and Vanquish Horizon UHPLC system. The analytical separation used an Accucore C30 column (2.1 mm × 150 mm, Thermo Fisher Scientific) with 50:50 acetonitrile/water and 88:10:2 isopropanol/acetonitrile/water, each containing 5 mM ammonium formate and 0.1% formic acid. LC-MS/MS data were acquired separately in positive and negative polarities with the following instrument parameters: MS1 scan 120,000 resolution; data-dependent MS2 on the top 20 most abundant ions at 15,000 resolution; 0.4 *m/z* isolation width; and a stepped normalized collision energy of 20:30:40 (43).

Lipids were identified and quantitated using LipidSearch 4.2 (Thermo Fisher Scientific). The identified lipid species were filtered by expected adducts and identification grade based on class. Peak areas were normalized to the EquiSPLASH standards for supported classes and further normalized based on total area for each sample to correct for variation in cell number. Statistical analysis was performed on log-transformed data using Perseus 1.6.2.3 (https://maxquant.net/perseus/) (41, 42).

### Metabolite extraction and analysis using LC-MS/MS for metabolome.

Polar metabolites were extracted from whole cells, Lyso-IP unbound fractions, and Lyso-IP bound samples using 80% methanol and were stored at –80°C prior to liquid chromatography–mass spectrometry (LC-MS) analysis. Extracts were analyzed by LC-MS using a Thermo Scientific Q-Exactive HF-X mass spectrometer with HESI II probe in-line with a Thermo Vanquish Horizon UHPLC system. LC separation was performed using a ZIC-HILIC column (2.1 mm × 150 mm, 5 μm particle size, EMD Millipore) with a ZIC-HILIC guard column (2.1 mm × 20 mm, EMD Millipore) held at 45°C with a flow rate of 0.2 mL/min. Chromatography was performed under acidic conditions to reduce reactivity of thiol groups. Mobile phase A was water and B was acetonitrile, both containing 0.1% formic acid. The LC gradient was 85% B for 2 minutes, 85% B to 20% B over 15 minutes, 20% B to 85% B over 0.1 minutes, and 85% B for 8.9 minutes. The autosampler was held at 4°C, and 4 μL of each sample was injected per analysis. The following MS parameters were used: sheath gas flow rate, 40 AU; auxiliary gas flow rate, 10 AU; sweep gas flow rate, 2 AU; auxiliary gas heater temperature, 350°C; spray voltage, 3.75 kV for positive mode and 3.5 kV for negative mode; capillary temperature, 375°C; and funnel RF level, 40%. All samples were analyzed by full MS with polarity switching. Full MS scans were acquired from 65 to 975 *m/z* at 120,000 resolution with an automatic gain control target of 1 × 10^6^ ions and maximum injection time of 100 milliseconds.

Raw data were analyzed using TraceFinder 4.1 (Thermo Fisher Scientific). Targeted metabolites were identified by accurate mass and retention time in their preferred polarity based on analytical standards. Peak detection used the ICIS algorithm with smoothing of 1. Relative quantification was performed using integrated peak area, and these values were corrected to total amount per sample (defined as total peak area).

### PPT1-CRISPR/Cas9 editing.

A375P *PPT1*-non-target and KO cells were prepared as previously described (44). pSpCas9(BB)-2A-Puro (PX459) was a gift from Feng Zhang (Addgene plasmid, 48139). Guide RNA oligos (IDT) were annealed, phosphorylated, and then ligated into *BbsI* digested and dephosphorylated pX459 plasmid following Zhang lab protocols, available through Addgene. Ligated plasmids were transformed into Stbl3 cells (Invitrogen). Sequencing of plasmid preps confirmed the presence of the desired guide RNA sequences. A375P cells were transfected using Lipofectamine 3000 (Invitrogen, L3000015), followed by puromycin selection. Surveyor assays confirmed editing of the PPT1 gene by CRISPR/Cas9, and *PPT1* knockdown was confirmed by Western blotting with PPT1 antibody (Origene). Sequences for guide RNA oligos are as follows: nontarget guide RNA forward (CACCGTAGCGAACGTGTCCGGCGT), nontarget guide RNA reverse (AAACACGCCGGACACGTTCGCTAC), human *PPT1* guide RNA 1 forward (CACCGTTTGGACTCCTCGATGCCC), human *PPT1* guide RNA 1 reverse (AAACGGGCATCGAGGGAGTCCAAA), human *PPT1* guide RNA 3 forward (CAACCGCCTCGTGCAAGCCGAATAC), and human *PPT1* guide RNA 3 reverse (AAACGTATTCGGCTTGCACGAGGC). The clones grown from single cells used in this paper include the following: clone C8 is human guide 1 and clone B5 is human guide 3.

### Immunoblotting.

One million cells were plated in a 10 cm dish, and treatments were given the next day; cells were collected by scraping. Whole-cell lysates were prepared using SDS lysis buffer. Protein concentration was measured using a Pierce BCA Protein Assay Kit (Thermo Fisher Scientific, 23225). 30–50 μg protein was used for SDS-PAGE and was transferred onto a PVDF membrane (Bio-Rad, 1620177). The membrane was blocked using 5% BSA or 5% skimmed milk, as per optimized conditions, and incubated with primary antibody overnight at 4°C. PVDF membranes were washed with 1× TBS-T (Cell Signaling Technology Inc., CS-9997) and incubated for 1 hour at room temperature with species-specific HRP-conjugated secondary antibody. Membranes were subsequently washed and developed using Pierce ECL Western Blotting substrate (Thermo Fisher Scientific, 32106) and autoradiography films (Lab Scientific Inc., XAR ALF 1318).

For pyroptosis studies, protein lysates were separated by SDS-PAGE and transferred to PVDF membranes. After blocking in 5% BSA, PVDF membranes were incubated with the indicated primary antibodies overnight at 4°C, washed in PBS/Tween, and incubated with peroxidase-coupled secondary antibodies. Immunoreactivity was detected using HRP-conjugated secondary antibodies (CalBioTech), chemiluminescence substrate (Thermo Fisher Scientific), and a ChemicDoc MP imaging system (Bio-Rad). For experiments involving supernatant, cells were cultured in FBS-free medium to avoid distortion of SDS-PAGE. Cell supernatants were harvested and centrifuged for 10 minutes at 500*g* at 4°C to remove cell debris. Resulting supernatants were concentrated 10× using Amicon Ultra 10K (MilliporeSigma) by centrifugation for 30 minutes at 4,500*g* at 4°C. Concentrates were mixed with sample buffer and analyzed via Western blotting as described above. Protein gel staining was performed using Ponceau red staining.

### LMP and cathepsin L activity assay.

Human pEGFP-hGal3 plasmid was a gift from Tamotsu Yoshimori (Addgene plasmid, 73080) and was used for creating the A375P-Galectin-3 line. pEGFP-C1 control plasmid vector backbone was purchased (novoprolabs, V012024). Plasmids were transfected (1 μg/mL) into A375P cells using Lipofectamine 2000 (Invitrogen, 11668019) per the manufacturer’s protocol; transfected cells were stably selected using G418 (Gibco, 10131035). For LMP, a total of 25 A375P-galectin-3 puncta–positive cells in multiple image fields were counted. The percentage of puncta-positive cells was calculated in each field separately, and an average percentage was calculated for each group. The Magic Red Cathepsin L assay kit (Abcam, ab270774) was used per the manufacturer’s protocol. Cells were imaged under a Zeiss Axio Observer 7 inverted microscope. Magic Red raw integrated intensity was calculated using Fiji - ImageJ software (NIH).

### MTT (3-[4, 5-dimethylthiazol-2-yl]-2, 5 diphenyl tetrazolium bromide) cell viability assay.

2,000 cells were plated in triplicates in each well of a 96-well plate, and 3-day MTT assays were performed using the Cell viability Kit (Roche, 11465007001) per the manufacturer’s protocol. Inhibitor pretreatment was given for 1 hour, followed by cotreatment of the cells with inhibitors with or without DC661. Nonlinear regression (curve fit) method was used to calculate IC_50_.

### Colony formation assay.

2,000 cells per well were seeded into a 6-well plate and were treated next day; cells were kept under the treatment for a total of 8 days. Colonies formed in each well were washed with PBS, fixed with cold methanol at –20°C for 20 minutes, and stained with Crystal violet 0.5% aqueous solution (V5265; MilliporeSigma).

### Trypan blue dye exclusion assay.

The reaction mixture for Trypan blue assay was prepared by mixing 1 part of 0.4% Trypan blue (25-900-CI, Corning) and 1 part of diluted cell samples. The mixture was incubated for 1 minute, and cells were counted on Cellometer Vision (Nexcelom, Vision-310-0208).

### FOAM-LPO.

LLP was studied using a fluorescent probe, FOAM-LPO. FOAM-LPO was synthesized as previously described (26). Cells were cultured on an 8 Well μSlide (ibidi, 80807) and treated with inhibitors and with or without DC661. Cells were incubated with media containing FOAM-LPO (1 M) in an atmosphere of 5% CO_2_ and 95% air for 5 minutes at 37°C. The cells were washed twice with media, and then cells were observed under the microscope (Zeiss Axio Observer 7 inverted microscope) to detect fluorescence shifting from 586 to 512 nm in response to LLP.

### qRT-PCR and primers.

A375P (3 × 10^5^) cells were cultured in 60 mm dishes and were treated with DMSO or DC661 (3 μM) for 24 hours. Total RNA was isolated with a RNA isolation kit (QIAGEN, 74134) according to the manufacturer’s protocol. cDNA was synthesized using an iScript Reverse Transcriptase kit with 500 ng of purified RNA as per manufacturer’s protocol (Thermo Scientific, K1642). The qPCR reaction was set up using SYBR Green PCR Master Mix (Bio-Rad, 1725121) containing 1 μL cDNA. All measurements were carried out in triplicate, and *BACTIN* was used as internal standard for ΔCT calculations. Gene expression analysis was done using the following primers: *PTGS2/COX-2* forward (CGGTGAAACTCTGGCTAGACAG), *PTGS2/COX-2* reverse (GCAAACCGTAGATGCTCAGGGA), *CARS* forward (CTGGACTACTCCAGCAACACCA), CARS reverse (GACCAGTGATGTCAACAGGAGC), *CHAC1* forward (GTGGTGACGCTCCTTGAAGATC), *CHAC1* reverse (GAAGGTGACCTCCTTGGTATCG), *BACTIN* forward (CAACTGGGACGACATGGAGAAAAT), and *BACTIN* reverse (CCAGAGGCGTACAGGGATAGCAC).

### siRNA transfection.

Genetic knockdown studies for human *ULK1*, *ATG7*, *TAX1BP1*, *BNIP3*, *PPT1*, and *MFSD12* were performed in A375P cell lines. Genetic inhibition studies for mouse *Ppt1* and *Calreticulin* were performed in MC38 cells. Nontarget siRNA (sc-37007), human *ULK1* (sc-44182), *ATG7* (sc-41447), *TAX1BP1*/*T6BP* (sc-106831), *BNIP3* (sc-37451), *PPT1*/*CLN1* (sc-105216), *MFSD12* (sc-97888), mouse *Ppt1*/*Cln1* (sc-142398) and *Calreticulin*/*Calregulin* (sc-29895) siRNAs were purchased from Santa Cruz Biotechnology. These siRNAs consist of pools of 3–5 target-specific siRNAs.

### Flow cytometry.

Ferroptosis induction was measured using C11-BODIPY (BODIPY 581/591 C11, Thermo Fisher Scientific, D3861). A375P cells were seeded in a 6-well plate and treated with DMSO, ferrostatin-1 (10 μM), liproxstatin-1 (2 μM), erastin (5 μM), DC661 (3 μM), or a combination for 24 hours. Cells were harvested by centrifuging at 300*g* for 5 minutes. Cells were resuspended in 500 μL 1X HBSS (Corning, 21-023-CV) containing 1 μM C11-BODIPY and incubated at 37°C for 15 minutes. Cells were pelleted and resuspended in 500 μL 1X HBSS, and fluorescence intensity was measured on a BD LSRII cytometer using 530/30 Blue (University of Pennsylvania Flow Core Facility).

Quantitation of cell surface expression of calreticulin for immunogenic cell death was performed as previously described (45) by flow cytometry. B16F10 or MC38 cells were cultured and treated with NAC (10 mM) or DC661 (3 μM) for 24 hours. MC38 cells were treated with si*Ppt1* or siNT for 48 hours in the presence or absence of 10 mM NAC for 24 hours. Cells were stained with CALR antibody (Cell Signaling Technology, 12238), Alexa Fluor 647 goat anti-rabbit second antibody (Invitrogen), and propidium iodide (PI) (Biolegend, 421301). Stained samples were acquired on a BD LSRII flow cytometer using 710/50 Blue and 670/30 Red to capture PI and CALR fluorescence, respectively (University of Pennsylvania Flow Core facility), and analysis was restricted to CALR-positive and PI-negative cells to avoid false-positive events.

### T cell priming and percentage cytotoxicity.

B16 or MC38 tumor cells (5 × 10^4^) were cultured and treated with NAC (10 mM) or DC661 (1 μM or 3 μM), respectively for, 24 hours. For *Calr* genetic inhibition, MC38 cells were treated with *Calr* siRNA or nontarget siRNA (siNT) for 48 hours, followed by treatment with either DMSO or 3 μM DC661 for 24 hours. Splenocytes were then cultured with DC661 with or without B16 or MC38 cells in the presence of IL-2 (5 IU/mL) and cocultured for 72 hours. Priming was confirmed by IFN-γ ELISA (Biolegend, 430815) of the supernatant. Primed splenocytes were then cocultured with freshly cultured B16 or MC38 cells with target-to-effector (B16 or MC38 to splenocytes) ratios of 1:20 and 1:50 for B16 and MC38 cells, respectively. The B16 or MC38 cell death–associated LDH release and then percentage cytotoxicity were measured according to the manufacturer’s protocol (MilliporeSigma, 4744926001) (31).

### Antitumor vaccination assays and chemotherapy studies with established cancer models.

Six- to eight-week-old female WT C57BL/6 mice, NOD/SCID γ, and BALB/c mice were obtained from The Jackson Laboratory. For antitumor vaccination experiments, WT B16F10, MC38, and CT26 cells were treated with DC661 (3 μM) for 36 hours in 175 cm^2^ flasks. Then, supernatants and detached cells were collected in a 50 mL falcon tube. The cells were centrifuged and washed with ice-cold PBS. For vaccination, 150 μL cellular suspension was injected s.c. in the left flank of immunocompetent C57BL/6 mice, immunodeficient NOD/SCID mice (1.8 × 10^4^ B16F10 cells, 1.5 × 10^6^ MC38 cells per mouse), or syngeneic BALB/c mice (3.0 × 10^6^ CT26 cells per mouse). Freeze-thawed cells resuspended in PBS were injected as a negative control. One week later, live cancer cells of the same types (3 × 10^4^ B16F10 cells, 2 × 10^5^ MC38 cells, or 5 × 10^5^ CT26 cells per mouse) with an equal volume of Matrigel (Corning, 354248) were injected in the right flank of vaccinated mice. Tumors were measured using electronic calipers, and volume was calculated as *L* × *W*^2^ × 0.5. Tumor growth was regularly monitored for the following days, and the absence of tumors was considered as an indication of efficient antitumor vaccination. DC661-MC38 vaccination studies were repeated in NOD/SCID mice.

### Statistics.

Statistical significance was determined by using the Student’s unpaired, 2-tailed *t* test when comparing 2 groups. The 1-way ANOVA test was used when more than 2 groups were compared. A null hypothesis was significantly rejected if the *P* value was less than 0.05. Data are shown as the mean ± SEM.

### Study approval.

All animal experiments were performed in accordance with the protocols approved by the Institutional Animal Care and Use Committee of the University of Pennsylvania.

## Author contributions

MB, DWS, and RKA conceived the project and designed the experiments. MB, AMV, and JJL performed the experiments. SLH and ARG performed the proteomics, lipidomics, and metabolomics study and analyzed the data. MASC prepared *PPT1* WT and KO A375P cell lines. MB, MASC, and VJ generated Galectin-3-EGFP-A375P cell lines. VJ generated Western blot data for TAX1BP1. MPS generated Western blot data for MHC class I and immunoproteasome activity. MV and AEA performed pyroptosis study and analyzed the data. SL, MM, and JDW synthesized FOAM-LPO. MB, JJL, AMV, and QL performed the statistical analysis. MB and RKA wrote the manuscript, and all authors reviewed the manuscript.

## Supplementary Material

Supplemental data

## Version 1 02/16/2023

In-Press Preview

## Version 2 04/17/2023

Electronic publication
