# Supplementary material for: Lysosomal lipid peroxidation regulates tumor immunity
Source: J Clin Invest. 2023 Apr 17;133(8):e164596. doi: 10.1172/JCI164596 (PMC10104903; doi:10.1172/JCI164596)
Supplement: Supplemental data [file jci-133-164596-s054.pdf]

Supplementary Figures

Supplementary Figure 1.

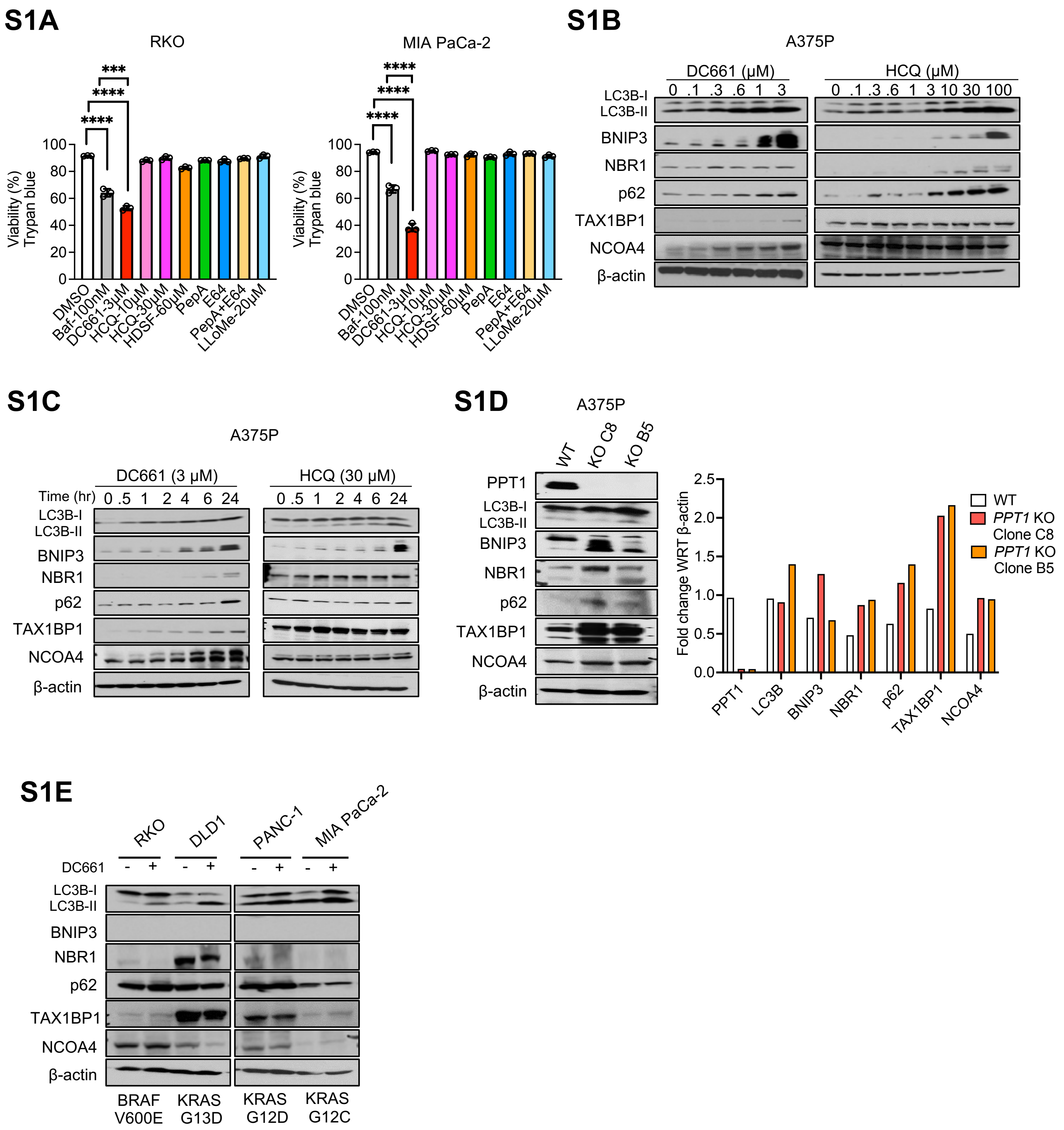

Supplementary Figure S1. Lysosomal autophagy inhibition induces significant changes in apoptosis and autophagy proteins. S1A. Trypan blue cell viability assay of colorectal RKO and pancreatic MIA PaCa-2 cancer cells treated with Bafilomycin-A1 (100 nM), DC661 (3 µM), HCQ (10 or 30 µM), HDSF (60 µM), Pepstatin A (10 µg/mL), E64 (10 µg/mL) or LLOMe (20 µM) for 48 hr. **S1B-S1C**. Immunoblots of LC3-I/II and autophagy cargo receptors in the whole cell lysates of A375P cells, treated with (S1B) indicated concentrations of DC661 or HCQ; (S1C) indicated timepoints with DC661 3 µM or HCQ 30 µM. **S1D-S1E.** Immunoblotting of whole cell lysates generated from (S1D) A375P WT and *PPT1* KO (clone C8 and clone B5) cells; (S1E) human RKO, DLD-1, Panc-1 and MIA PaCa-2 cells treated with DC661 3 µM for 24 hr. ***P ≤ 0.001; ****P ≤ 0.0001. ANOVA test was used when more than two groups were compared.

Supplementary Figure 2.

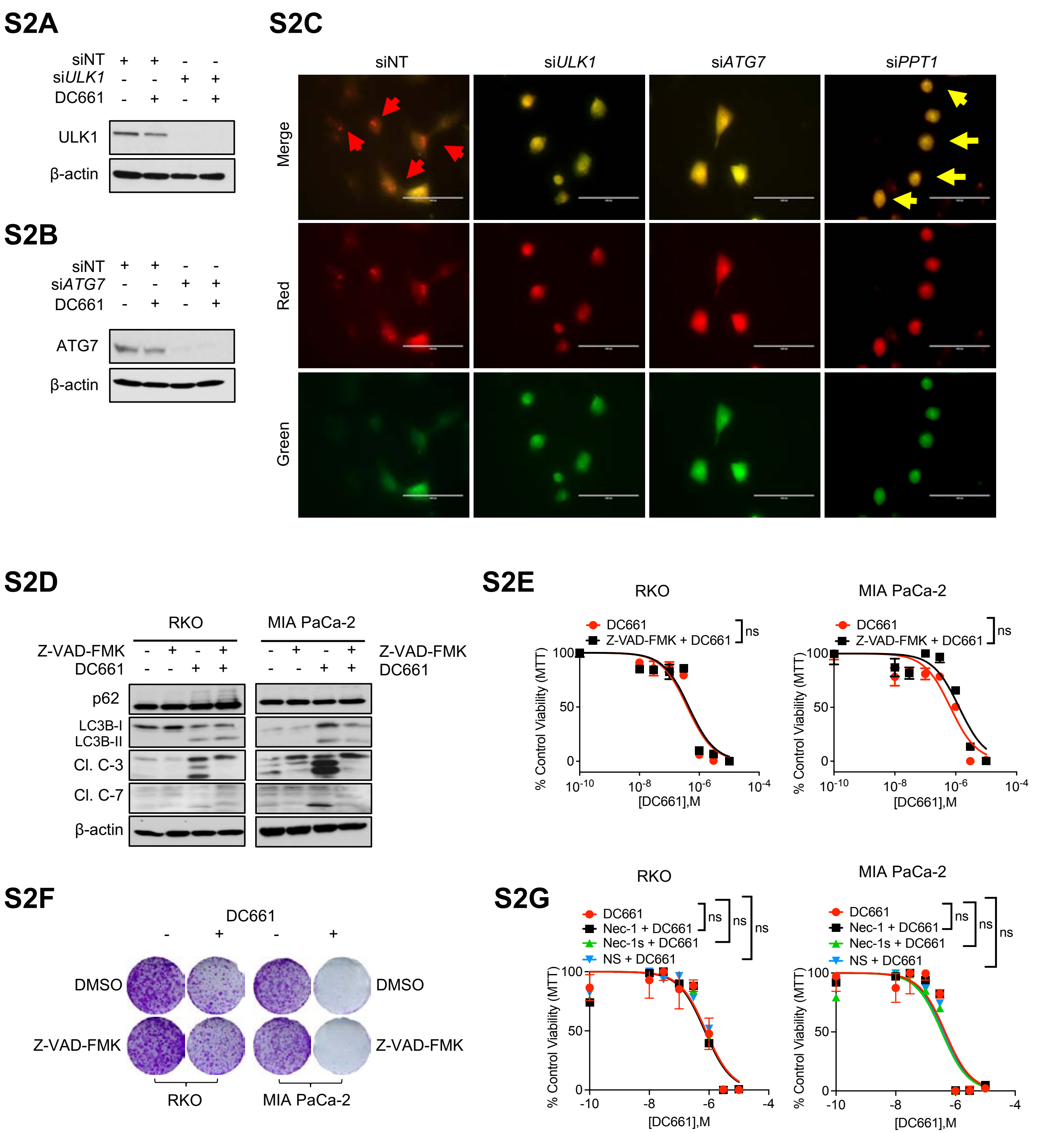

Supplementary Figure S2. DC661-induced apoptosis and necroptosis. S2A-S2B. A375P cells were transfected with non-target siRNA (siNT) or si*ULK1* or si*ATG7* for 48 hr followed by treatment with either DMSO or DC661 3 μM for 24 hr. Lysates were immunoblotted for (S2A) ULK1 or (S2B) ATG7. **S2C**. A375P cells expressing mCherry-eGFP-LC3B, were transfected with siNT, si*ULK1*, si*ATG7*, or si*PPT1* for 24 hr followed by fluorescent microscopy imaging. Scale bar = 40 μm **S2D.** Immunoblots of LC3-II, p62, Cleaved caspase 3 (Cl. C-3) and cleaved caspase 7 (Cl. C-7) in the lysates of RKO and MiaPaCa2 treated with DC661 3 μM, Z-VAD-FMK 80 μM or both for 24 hr. **S2E.** 72 hr MTT assay in cells treated with DC661 (0.01 to 10 μM), with and without Z-VAD-FMK 80 μM. **S2F.** 7-day colony formation assay in cells treated with DC661 0.3 μM, Z-VAD-FMK 1 μM or the combination. **S2G.** 72 hr MTT assay in cells treated with DC661 (0.01 to 10 μM), with and without necrostatin-1 (Nec-1, 50 μM), necrostatin 1s (Nec-1s; 50 μM) and necrosulfonamide (NS; 2.5 μM) in RKO and MiaPaCa2 cells. ns: non-significant. Two-tailed unpaired t test between two groups (Figure S2E). ANOVA test was used when more than two groups were compared.

Supplementary Figure 3.

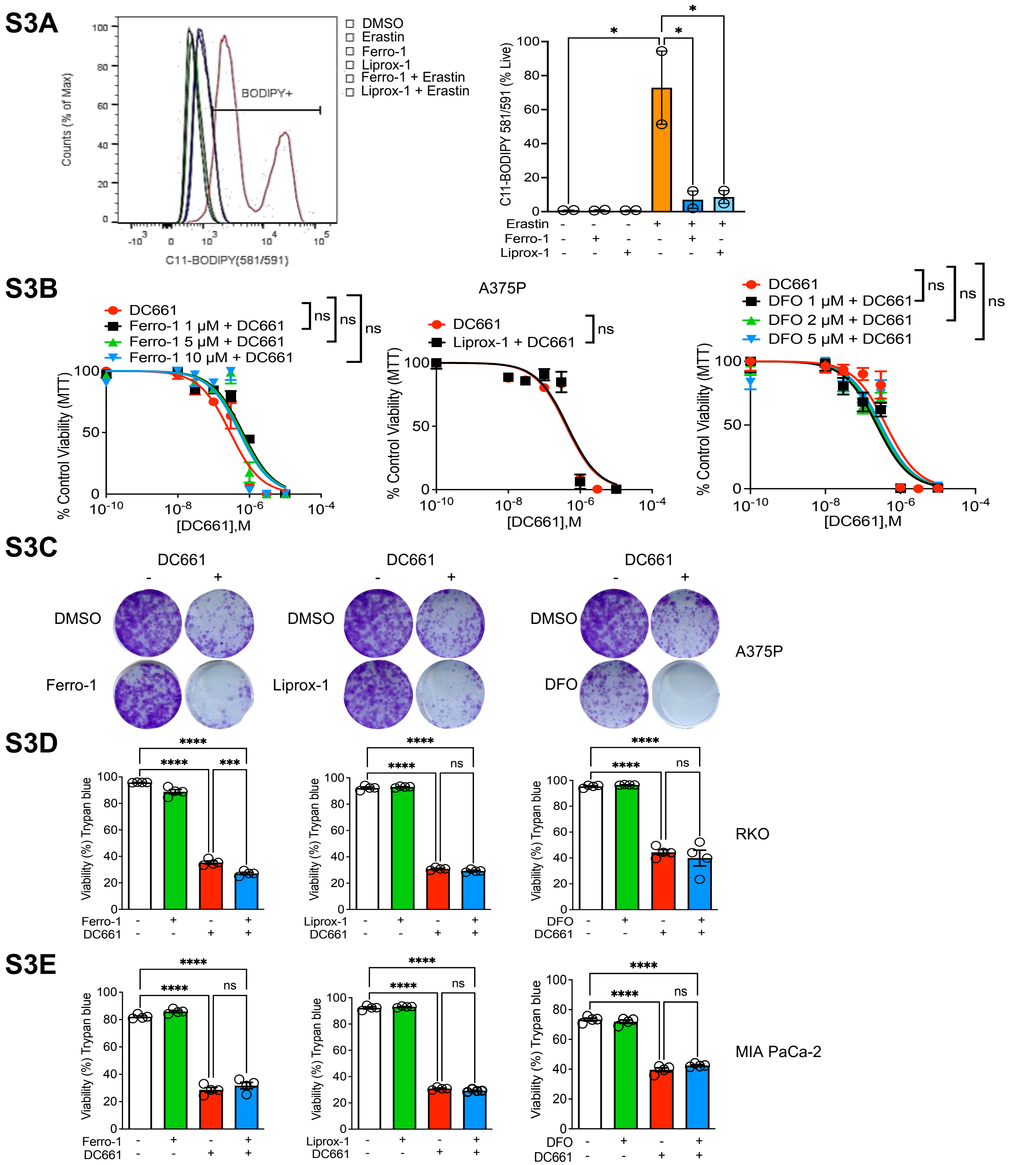

Supplementary Figure S3. DC661-induced ferroptosis. S3A. A375P cells treated for 24 hr with Erastin (5 μM), Liproxstatin-1 (Liprox-1, 2 μM) or Ferrostatin-1 (Ferro-1, 10 μM). Lipid peroxidation measured by C-11 BODIPY using flow cytometry. The single agent controls (DMSO, Ferro-1, Liprox-1) are same experiment as Figure 3B. **S3B**. 72 hr MTT assay in cells treated with DC661 (0.01 to 10 μM), with and without indicated concentrations of Ferro-1, Liprox-1 2 μM and iron chelator desferoxamine (DFO) in A375P cells. **S3C.** 7-day colony formation assay in A375P cells treated with DC661 0.3 μM, Ferro-1 10 μM, Liprox-1 2 μM, DFO 5 μM or combinations. The same DMSO control is shown in all 3 subpanels in S3C. **S3D-S3E.** Trypan blue cell viability assay in RKO (S3D) and MIA PaCa-2 (S3E) cells treated with DC661 3 μM, Ferro-1 10 μM, Liprox-1 2 μM, DFO 5 μM or both for 72 hr. **P ≤ 0.01; ***P ≤ 0.001; ****P ≤ 0.0001; ns: non-significant. Two tailed unpaired t-test between two groups (Figure S3B; DC661 and Liprox-1 +DC661). ANOVA test was used when more than two groups were compared.

Supplementary Figure 4.

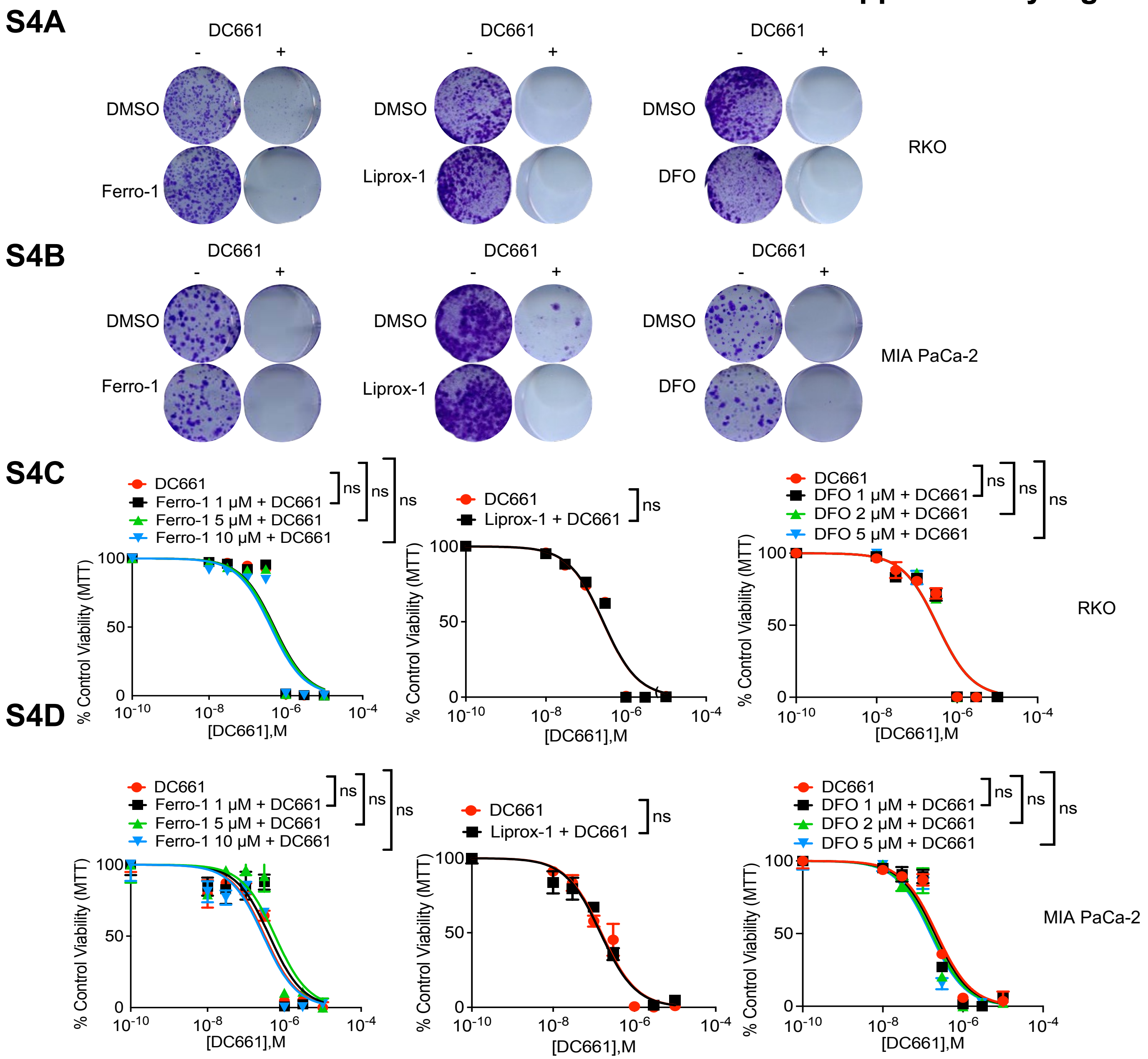

Supplementary Figure S4. DC661 induced- ferroptosis. S4A-S4B. 7-day colony formation assay in RKO (S4A) and MIA PaCa-2 (S4B) cells treated with DC661 0.3 μM, Ferrostatin-1 (Ferro-1 10 μM), Liproxstatin-1 (Liprox-1 2 μM), desferoxamine (DFO 5 μM) or their combinations. **S4C-S4D**. 72 hr MTT assay plot with increasing concentrations of DC661 (0.01 to 10 μM), with and without indicated concentrations of Ferro-1, DFO and Liprox-1, 2 μM in RKO (S4C) and MIA PaCa-2 (S4D) cells. ns: non-significant. Two tailed unpaired t-test between two groups (Figures S4B and S4D; DC661 and Liprox-1 +DC661). ANOVA test was used when more than two groups were compared.

Supplementary Figure 5.

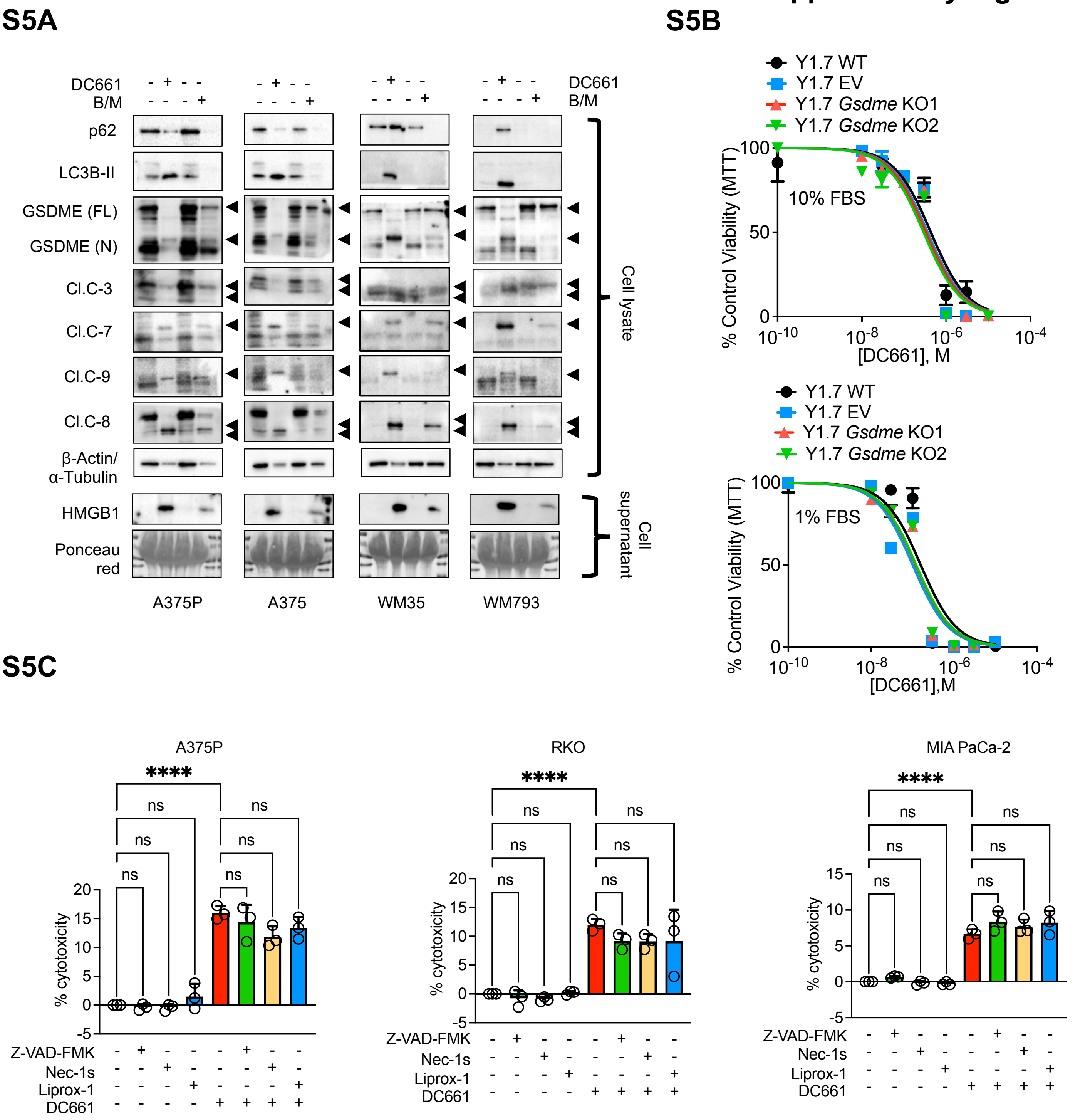

Supplementary Figure S5. DC661-induced pyroptosis. S5A. Western blots probed for pyroptosis and autophagy proteins in the whole cell lysates and HMGB1 release in cell supernatant of human melanoma A375P, A375, WM35 and WM793 cells treated with DC661 1 μM and pyroptosis inducers BRAF inhibitor PLX4720 1 μM (B) and MEK inhibitor PD0325901 35 nM (M) for 48 hr. **S5B.** 72 hr MTT assay in in mouse YUMM 1.7 empty vector (EV), gasdermin-E knockout 1 and 2 (*Gsdme*-KO1 and KO2) cells treated with DC661 (0.01 to 10 μM) in both 10% and 1% FBS conditions. **S5C.** LDH release assay in cells treated with DC661 and Z-VAD-FMK (80 μM), Necrostatin-1s (Nec-1s, 50 μM), or Liproxstatin (Liprox-1, 2 μM). ***P ≤ 0.001; ns: non-significant. ANOVA test was used when more than two groups were compared.

Supplementary Figure 6.

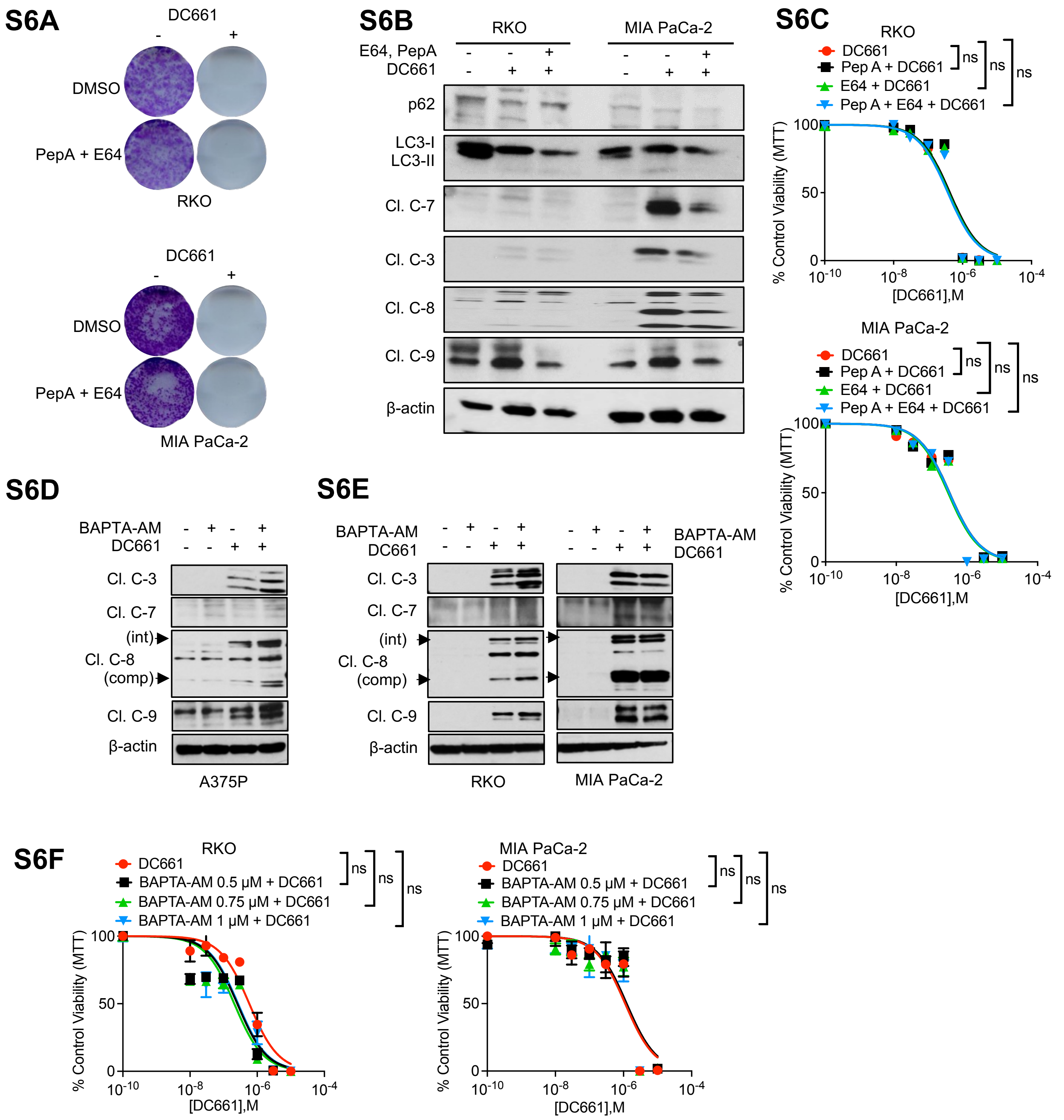

Supplementary Figure 6. Cathepsin inhibition or calcium chelation does not prevent DC661-induced cell death. S6A. 7-day colony formation assay in cells treated with PepA + E64 with or without DC661 0.3 μM. **S6B.** immunoblots for cleaved caspase3 (cl. C3), -7 (cl. C7), -8 (cl. C8), -9 (cl. C9), LC3I/II and p62 in cell lysates treated with Pepstatin A (PepA,10 μg/ml) + E64 10 μg/ml with or without DC661 3 μM for 24 hr. **S6C.** 72 hr MTT assay in cells treated with DC661 (0.01 to 10 μM) with or without PepA, E64 and PepA +E64. **S6D-S6E.** immunoblotting for caspases was performed with the lysates of cells treated with DC661 3 μM, BAPTA-AM 1 μM or both for 24 h. **S6F.** 72 hr MTT assay of cells treated with DC661 (0.01 to 10 μM), with and without indicated concentrations of BAPTA-AM. ns: non-significant. ANOVA test was used when more than two groups were compared.

Supplementary Figure 7.

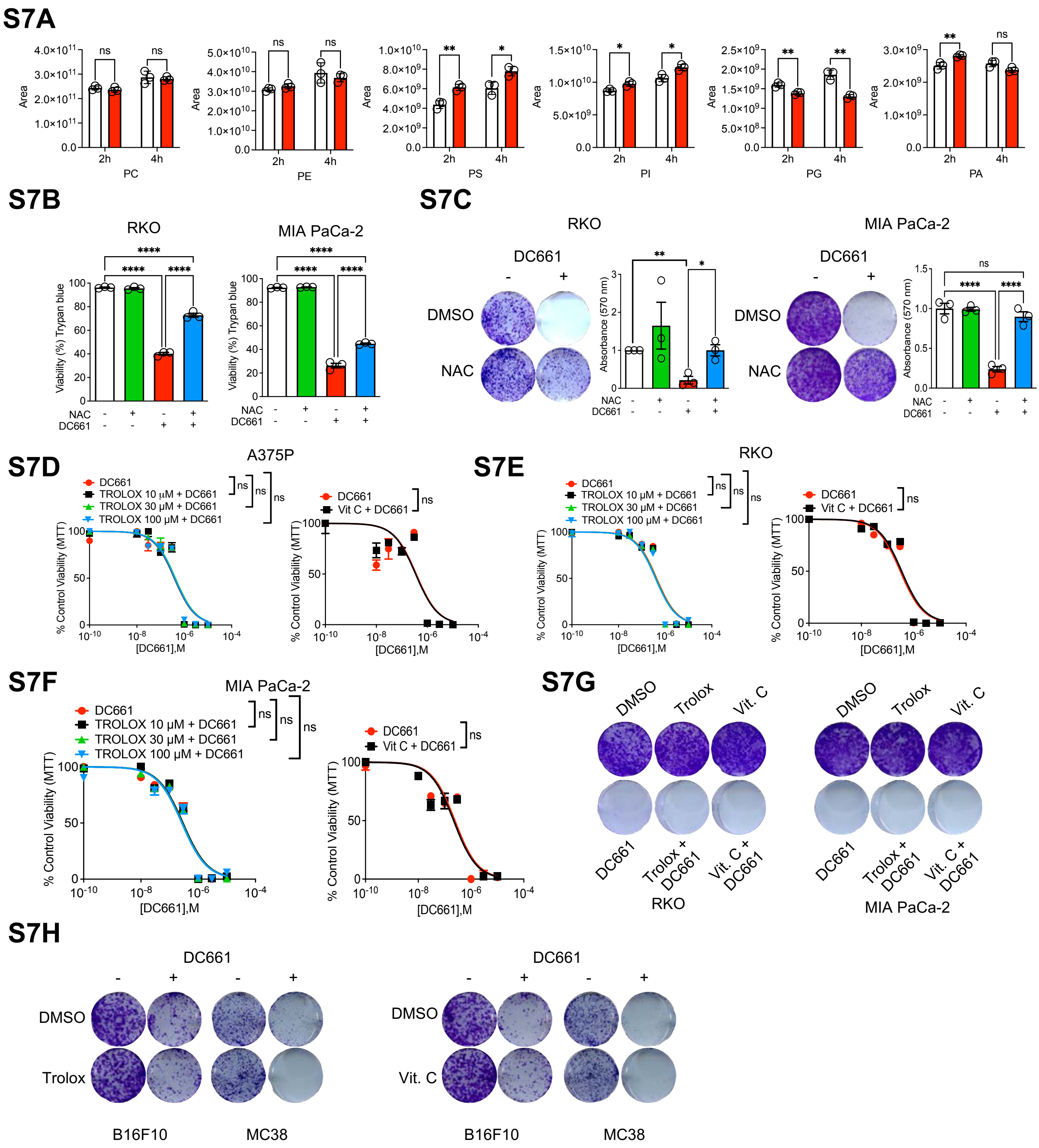

Supplementary Figure S7. N-acetyl cysteine (NAC) prevents DC661-induced cell death. S7A. LC-MS/MS lipidome analysis of lysosomes purified from A375P cells treated with DMSO (white) or DC661 3 μM (red), for 2 or 4 hr. Mean +/- SD of significantly elevated lipid classes. PC: phosphatidyl choline, PE: phosphatidyl ethanolamine, PS: phosphatidyl serine, PI: phosphatidyl inositol, PG: phosphatidyl glycerol and PA: phosphatidyl acid. **S7B.** Trypan blue viability of cells treated with NAC 10 mM, DC661 3 μM or both. **S7C.** 7-day colony formation assay in cells treated with DC661 0.2 μM, NAC 1 mM or both. **S7D-S7F.** 72 hr MTT assay in cells treated with DC661 (0.01 to 10 μM), with and without indicated concentrations of Trolox and Vitamin C (Vit. C; 100 μM). **S7G- S7H.** 7-day colony formation assay in RKO, MIA PaCa-2, B16F10 and MC38 cells treated with DC661 0.2 μM, Trolox 100 μM, Vitamin C 100 μM or in combinations. *P ≤ 0.05; **P ≤ 0.01; ***P ≤ 0.001; ****P ≤ 0.0001; ns: non-significant. Two-tailed unpaired t test between two groups (Figure S7A; Figure S7C RKO; Figures S7D-S7F, DC661 and Vit C + DC661). ANOVA test was used when more than two groups were compared.

Supplementary Figure 8.

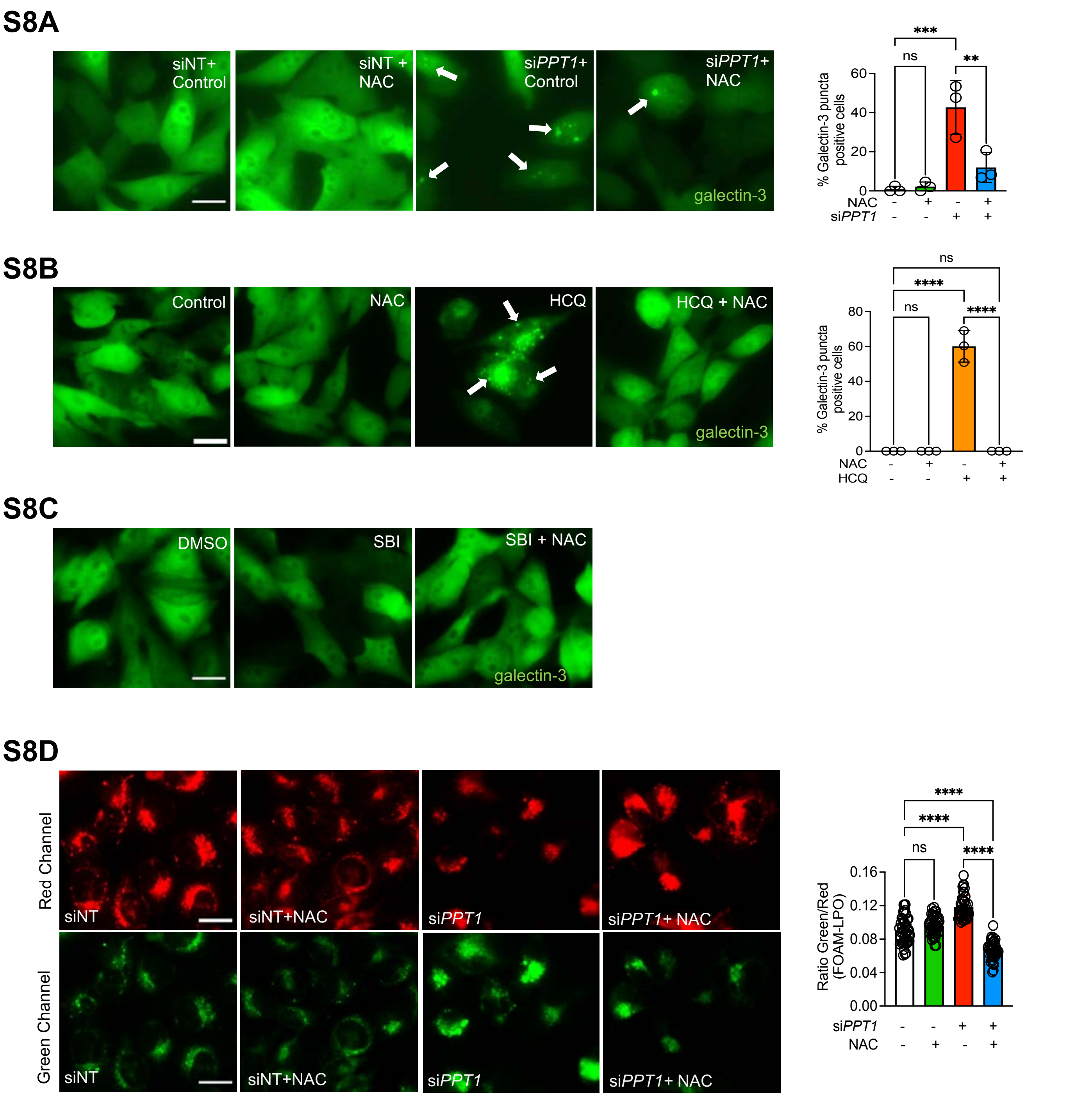

Supplementary Figure S8. N-acetyl cysteine (NAC) rescues PPT1 inhibition. S8A-C. A375P-galectin-3-GFP cells were used to measure lysosomal membrane permeabilization (white arrows). **S8A.** treated with si*PPT1* or siNT for 48 hr, in the presence or absence of NAC 10 mM for 24 hr. **S8B.** treated with HCQ 100 µM and NAC 10 mM for 48 hr. **S8C.** treated with ULK1 inhibitor SBI0206965 (SBI; 3 µM) alone or in combination with NAC 10mM. **S8D.** Fluorescence images of A375P cells stained with Foam-LPO (1 µM, 5 min) to detect lysosomal lipid peroxidation. Scale bar = 20 µm. *P ≤ 0.05; **P ≤ 0.01; ***P ≤ 0.001; ****P ≤ 0.0001; ns: non-significant. ANOVA test was used when more than two groups were compared.

Supplementary Figure 9.

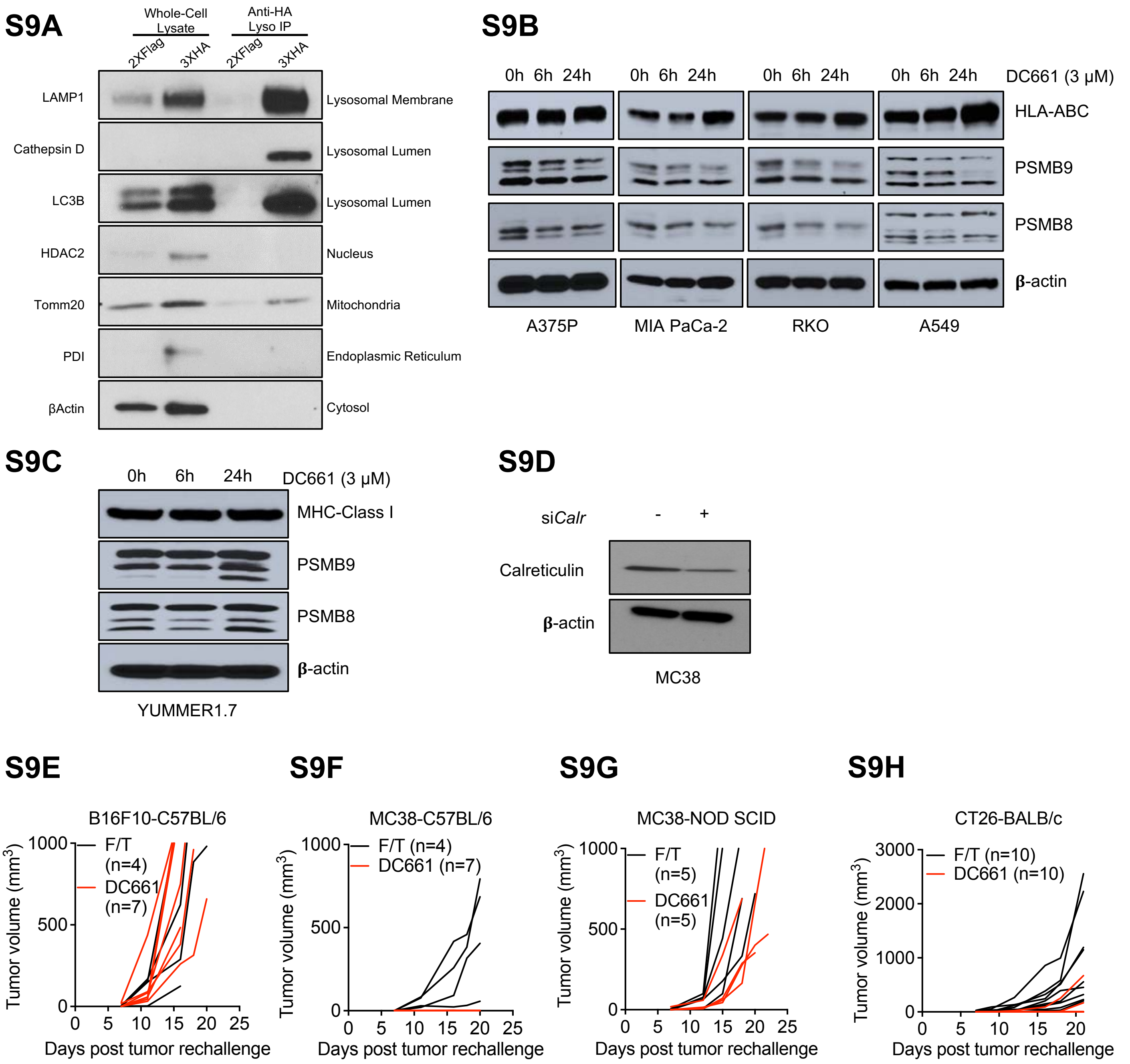

Supplementary Figure S9. The LysoIP method isolates pure lysosomes and anti-cancer vaccination effects of DC661. S9A. Immunoblotting for protein markers of various subcellular compartments in whole cell lysates, purified lysosomes (3XHA Lyso IP), or control immunoprecipitants (2xFlag). Lysates were prepared from cells expressing the 2xFlag-tagged TMEM192 (Control-Lyso cells) or 3xHA-tagged Tmem192 (HA-Lyso cells). **S9B-S9C.** immunoblots of the lysates of human and murine cells treated with indicated concentrations of DC661 for 6 and 24 hr. **S9D.** Immunoblotting for calreticulin and **β**-actin of MC38 cells that were treated with calreticulin (CALR) siRNA or non-target siRNA (siNT) for 48 hr. **S9E-S9H.** Cells were treated with DMSO or DC661 for 36 hr, then subcutaneously injected (1.8×10^5 B16F10 cells, 1.5×10^6 MC38 cells, or 3.0×10^6 CT26 cells per mouse) into the left flank of immunocompetent syngeneic C57BL/6J, BALB/c mice, or immunodeficient NOD-SCID mice. Freeze-thawed (F/T) DMSO treated cells was used as control. One week later all mice were rechallenged and subcutaneously injected with live untreated cells (3×10^4 B16F10 cells, 2×10^5 MC38, or 5×10^5 CT26 cells per mouse) into the right flank of corresponding vaccinated mice. Spaghetti plot of tumor volumes for each individual mouse in each treatment group shown. n = 4-10 per group.
